# Pan-cancer multi-omics profiling of OAS3 reveals its immunological and prognostic associations across human cancers

**DOI:** 10.7717/peerj.20805

**Published:** 2026-02-12

**Authors:** Xi Zhang, Hongyan Zhang, Liang Zhong, Wei Yang, Chunhui Yang, Yuqing Pan, Beizhong Liu

**Affiliations:** 1 Central Laboratory of Yong-Chuan Hospital, Chongqing University of Medical Science, Chongqing, China; 2 Key Laboratory of Laboratory Medical Diagnostics, Chongqing University of Medical Science, Chongqing, China; 3 Department of Clinical Laboratory, Caner Hospital of Yunnan Province, The Third Affiliated Hospital of Kunming Medical University, Kunming Medical University, Kunming, China; 4 Yunnan Key Laboratory of Laboratory Medicine, Kunming Medical University, Kunming, China; 5 Clinical Laboratory of the Affiliated Rehabilitation Hospital, Chongqing University of Medical Science, Chongqing, China

**Keywords:** OAS3, Pan-cancer, Immune modulation, Prognostic biomarker, Immune infiltration

## Abstract

**Background:**

The enzyme 2′-5′-oligoadenylate synthetase 3 (OAS3) plays a well-established role in antiviral immunity, but its involvement in cancer biology remains poorly understood. This study aimed to comprehensively investigate the expression patterns, mutation characteristics, prognostic significance, functional roles, and therapeutic potential of OAS3 across multiple cancer types.

**Methods:**

Utilizing multi-omics data from public cancer databases, we analyzed OAS3 expression patterns, survival correlations, immune interactions, pathway enrichment, genomic associations, mutation profiles, and functional validation of OAS3 knockdown in THP-1 cells for apoptosis assays.

**Results:**

OAS3 expression was significantly upregulated in 32 cancer types, highlighting its context-dependent roles as an oncogene. The expression patterns of OAS3 were found to be stage-specific and cancer-specific across multiple tumor types. Survival analysis revealed that high OAS3 expression was significantly associated with unfavorable prognosis in several cancers. Further analyses demonstrated that OAS3 expression was correlated with immune-related genes, immune checkpoints, tumor stemness, and immune cell infiltration across multiple tumor types. Moreover, gene set enrichment profiling revealed significant involvement of OAS3 in interferon response and immune regulation, with prominent enrichment in Janus kinase-signal transducer and activator of transcription (JAK-STAT) and Notch signaling pathways. Mutation analysis highlighted high mutation frequencies of OAS3 in several cancer types, especially in endometrial cancer and melanoma. Comprehensive molecular profiling further identified significant associations between OAS3 expression levels and key genomic features (tumor mutation burden, homologous recombination deficiency and RNA modification-related proteins). Besides, OAS3’s expression was associated with key processes such as cell cycle regulation and immune evasion, further underscoring its potential as a therapeutic target in lung and breast cancers. Finally, we found that OAS3 was observably related with some mutation types (CEBPA, FLT3 internal tandem duplication, NRAS, and EVI1 expression) in patients with acute myeloid leukemia (LAML). Functional validation through RNA interference demonstrated that OAS3 knockdown significantly induced apoptosis in THP-1 cells.

**Conclusion:**

This study demonstrates that OAS3 acts as a pivotal modulator in the complex network of cancer progression, highlighting its dual role in both tumorigenesis and immune response regulation.

## Introduction

Cancer constitutes a heterogeneous group of malignancies driven by cumulative genomic alterations that disrupt cellular homeostasis, culminating in uncontrolled proliferation and functional dedifferentiation (Hanahan & Weinberg, 2000; Hanahan & Weinberg, 2011). These transformed cells employ sophisticated immune evasion mechanisms to bypass host surveillance, subsequently establishing primary neoplasms and metastatic lesions (Shi et al., 2024). Epidemiological data reveal substantial heterogeneity in cancer prevalence, with pulmonary, mammary, and colorectum carcinomas representing predominant clinical entities across global populations (Bray et al., 2024). Contemporary therapeutic paradigms integrate multimodal strategies: surgical resection serves as the cornerstone for localized disease control, whereas cytotoxic chemotherapy and ionizing radiation therapy modulate disease progression through the induction of apoptosis and DNA damage response. Emerging biological therapies, particularly immune checkpoint inhibitors and monoclonal antibodies, harness adaptive immunity to recognize tumor-specific antigens (Yadav et al., 2025). Parallel advancements in molecularly targeted agents enable precise interception of oncogenic signaling cascades. Notably, the advent of precision oncology has catalyzed the development of biomarker-guided combinatorial regimens (Rosenblum et al., 2018), strategically synergizing these modalities to optimize therapeutic efficacy while mitigating systemic toxicity.

Hence, identifying molecular targets that coordinate both oncogenic signaling and immune evasion mechanisms has become a pivotal frontier in cancer research. 2′-5′-oligoadenylate synthetase 3 (OAS3) is a pivotal enzyme within the innate immune system, primarily involved in antiviral responses through the activation of RNA degradation pathways and the induction of interferon-stimulated genes (Kristiansen et al., 2011; Lohöfener et al., 2015). Originally characterized as a cytoplasmic sensor mediating antiviral responses through RNase L activation, emerging evidence implicates OAS3 in oncogenic reprogramming, particularly through its immunomodulatory functions (Zhang et al., 2024), tumor-promoting activities (Shi et al., 2022), and contributions to therapeutic resistance mechanisms (Caglar et al., 2024). Despite its potential as a therapeutic target, a comprehensive understanding of OAS3’s multifaceted role in cancer biology remains limited, especially across different cancer types. Pan-cancer research, which focuses on understanding genes that contribute to cancer development and progression across a wide range of tumor types, has gained significant traction in recent years (Adashek et al., 2024). As part of the emerging field of pan-cancer genomics, the study of genes like OAS3, which are involved in multiple cancers, can reveal shared molecular mechanisms that govern cancer biology and prognosis (Li et al., 2022b). OAS3’s expression levels, mutations, and its interactions within the tumor microenvironment offer valuable insights into its potential as a diagnostic and prognostic biomarker.

This study aims to explore OAS3 in the context of pan-cancer, analyzing its gene expression profiles, mutation characteristics, and the associated immune infiltrates across various cancer types. By utilizing data from major cancer databases, we will examine how OAS3’s expression correlates with patient survival, immune response, and key cancer signaling pathways. Additionally, we will investigate how modulating OAS3 expression may influence cancer treatment outcomes, particularly in terms of immune evasion and tumor stemness. This research focuses on highlighting the therapeutic potential of targeting OAS3 in cancer treatment, underlining its importance as a promising candidate for precision medicine strategies aimed at improving cancer prognosis and treatment efficacy.

## Materials & Methods

### Data collection

In this study, we utilized the pan-cancer dataset from the UCSC Xena Browser (https://xenabrowser.net/), which integrates harmonized data from multiple large-scale cohorts, including The Cancer Genome Atlas (TCGA), Therapeutically Applicable Research to Generate Effective Treatments (TARGET), and Genotype-Tissue Expression (GTEx). The dataset comprises 19,131 samples and expression profiles for 60,499 genes. We specifically focused on the expression data of the OAS3 gene (ENSG00000111331) across all available samples. To normalize the data and mitigate potential biases introduced by distributional skewness, we applied a log2 transformation with a pseudocount of 0.001 (log2(x + 0.001)) to each expression value. Genes with zero expression values across all samples were removed prior to downstream analyses to reduce noise from non-expressed features. In addition, cancer types with fewer than three available samples were excluded to ensure that only well-represented cancer subtypes were retained for subsequent analyses. The abbreviations of the cancer types were listed in Table S1.

### Differential expression analysis

Differential expression of OAS3 between normal and tumor samples within each cancer type was analyzed using R software. Additionally, we examined the mRNA expression levels of OAS3 in normal human tissues using the Human Protein Atlas (HPA) database (https://www.proteinatlas.org) and assessed protein expression in normal/tumor tissues and tumor cell lines through immunohistochemistry (IHC) and immunofluorescence (IF) techniques.

### Pathological stage analysis of OAS3 across various cancer types

The “Stage plot” feature from the Gene Expression Profiling Interactive Analysis (GEPIA2, http://gepia.cancer-pku.cn/) database was used to examine how OAS3 expression correlates with tumor stages across various cancer types. Furthermore, to investigate the OAS3 expression variation across various clinical stages in different cancer types, we utilized the limma package in R to analyze the TCGA pan-cancer dataset. Expression levels of OAS3 were compared across different clinical stages within each cancer type.

### Proportional hazards regression and survival time analysis

In this investigation, we leveraged the prognostic data from TCGA obtained from previous research (Liu et al., 2018), supplemented by follow-up information from the TARGET dataset obtained through UCSC. To ensure the reliability of the survival data, samples with follow-up durations of less than 30 days were excluded. Transcriptomic data preprocessing involved a log2 transformation (log2(x+0.001)) to normalize expression distributions, with cancer types having fewer than 10 samples excluded to ensure statistical reliability. Clinical correlation analyses were conducted using Cox proportional hazards regression modeling through the coxph function from the R package survival (v3.2.7) to examine the association between OAS3 expression levels and patient outcomes. Prognostic significance was evaluated across 44 cancer types, with statistical testing performed using the Log-rank test. Additionally, survival analyses for both overall survival (OS) and disease-free survival (DFS) were conducted using the GEPIA2 platform.

### OAS3 expression correlation with immune genes, checkpoints, and stemness scores

We extracted the expression data of OAS3 from the TCGA Pan-Cancer dataset and, based on relevant literature (Hu et al., 2021), compiled the expression data for 41 cytokine signaling genes, 18 receptor genes, 21 major histocompatibility complex (MHC) genes, 24 immune inhibitory molecule genes, and 46 immune-stimulatory molecule genes across the samples. Normal samples were excluded from the analysis to reduce their influence. We then conducted Spearman’s rank-order correlation to explore the association between OAS3 expression and the expression of five categories of immune-related genes. Additionally, we retrieved the expression data for 24 inhibitory immune checkpoint genes and 36 stimulatory immune checkpoint genes (Hu et al., 2021), and calculated the Spearman correlation between OAS3 expression and these two categories of immune checkpoint genes. Finally, tumor stemness scores for each cancer type were obtained from related studies (Malta et al., 2018), calculated based on methylation features. By integrating the stemness scores and gene expression data, we computed the Spearman correlation between OAS3 expression and stemness scores in each cancer type.

### Immune feature analysis

Tumor microenvironment characterization was performed using the TARGET GTEx pan-cancer dataset. ESTIMATE scores were computed for individual samples through the ESTIMATE algorithm (R package v1.0.13) to quantify tumor microenvironment composition. Correlation analyses between OAS3 expression patterns and immune infiltration metrics were conducted using corr.test function (R package psych v2.1.6). Furthermore, gene expression data from all TCGA cancer types were obtained, and immune infiltration levels for immune cell types were calculated using the TIMER2 platform (http://timer.cistrome.org/). Immune cell infiltration was further evaluated using the IOBR R package (version 0.99.9) (Zeng et al., 2021). Specifically, the deconvo_xCell function (Aran, Hu & Butte, 2017) and the deconvo_CIBERSORT function (Newman et al., 2015) were applied to estimate immune cell fractions across TCGA/TARGET cohorts.

### OAS3-related gene analysis

The protein-protein interaction (PPI) centered on OAS3 was initially reconstructed using the STRING database (v11.5, https://string-db.org/), enabling identification of high-confidence interactors and their functional annotations in cellular pathways. Subsequent transcriptomic correlation analyses identified the top 100 OAS3-associated genes through the GEPIA2 platform, with detailed results provided in Table S2. These candidate genes were further validated *via* the TIMER2.0 computational framework to assess pan-cancer expression correlations. Functional annotation of OAS3-related networks was performed through Gene Ontology (GO) biological processes and Kyoto Encyclopedia of Genes and Genomes (KEGG) pathways, implemented in the Sangerbox 3.0 platform (false discovery rate (FDR) < 0.05, http://www.sangerbox.com) (Shen et al., 2022).

### Association of OAS3 with genetic alteration in pan-cancer

The genetic alteration patterns of OAS3 across various cancer types were examined through the cBioPortal platform (http://www.cbioportal.org/). Subsequently, homologous recombination deficiency (HRD) data for each cancer type, obtained from previous studies, were integrated into the analysis. Cancer types having fewer than three samples were excluded to ensure statistical analysis. Additionally, gene expression data for 10 genes related to m1A modification, 15 genes related to m5C modification, and 26 genes associated with m6A modification were extracted from the standardized TCGA Pan-Cancer dataset. Furthermore, the simple nucleotide variation (SNV) dataset was downloaded from the Genomic Data Commons (GDC) portal (https://portal.gdc.cancer.gov/), and tumor mutational burden (TMB) for each cancer type was calculated using the maftools package in R language. Finally, the correlation between OAS3 expression, HRD, and TMB was assessed to explore the potential role of OAS3 in genetic alterations across pan-cancer.

### Cellular characteristics and functional analysis of OAS3 in tumors

We utilized Cancer Single-cell Expression Atlas (CancerSEA, http://biocc.hrbmu.edu.cn/CancerSEA/) to analyze the functional characteristics of OAS3 in tumor cells, focusing on key processes such as DNA damage, invasion, and cell cycle regulation. Additionally, we employed LinkedOmics (https://www.linkedomics.org) to identify gene networks associated with OAS3 in lung cancer and breast cancer, exploring the interactions between OAS3 and other genes and their potential mechanisms in cancer.

### OAS3 expression and prognostic mutations in acute myeloid leukemia (LAML)

The correlation between OAS3 expression and several key mutations associated with prognosis in LAML was analyzed using the KMplot database (https://kmplot.com/analysis/). Specifically, survival curves of patients with low and high OAS3 expression levels were examined in relation to mutations in isocitrate dehydrogenase 1 (IDH1), isocitrate dehydrogenase 2 (IDH2), Nucleophosmin 1 (NPM1), CCAAT enhancer binding protein alpha (CEBPA), FMS-like tyrosine kinase 3 internal tandem duplication (FLT3-ITD), FMS-Like tyrosine kinase 3 tyrosine kinase domain (FLT3-TKD), neuroblastoma RAS viral oncogene homolog (NRAS), and ecotropic virus integration site 1(EVI1).

### Cell culture and transfection

THP-1 cells (Cat. No. TIB-202, ATCC, USA) were cultured as the model for OAS3 gene knockdown. OAS3 silencing was achieved using si-OAS3 (5′-GGAAGGAGTTCGTAGAGAA-3′), purchased from Genechem (Shanghai, China). Transfection was performed using RFectSP siRNA Transfection Reagent (Cat. No. 11026, Baidai Biotechnology, China), following the manufacturer’s instructions. Cells were divided into three groups: an OAS3 knockdown group (si-OAS3), a negative control group (si-NC), and an untreated group (UG). Following transfection, cells were cultured in RPMI-1640 medium (Cat. No. 11879020, Gibco) supplemented with 10% fetal bovine serum (FBS) (Cat. No. A5670701, Gibco) at 37 °C in a 5% CO_2_ incubator.

### Western blotting analysis

THP-1 cells were lysed in radioimmunoprecipitation assay (RIPA) buffer (Cat. No. R0010, Solarbio, Beijing, China), and protein concentration was measured using the bicinchoninic acid (BCA) assay (Cat. No. P0010, Beyotime, China). Equal amounts of protein (30 µg) were separated by sodium dodecyl sulfate–polyacrylamide gel electrophoresis (SDS-PAGE) and transferred to a polyvinylidene fluoride (PVDF) membrane (Cat. No. 03010040001, Millipore, MA, USA). After blocking, membranes were incubated overnight with primary antibodies against OAS3 (1:2,000, Cat. No. ab154270, Abcam, Cambridge, UK) and glyceraldehyde-3-phosphate dehydrogenase (GAPDH) (1:1,000, Cat. No. AF1186, Beyotime, China), followed by HRP-conjugated goat anti-rabbit secondary antibodies (1:1,000, Cat. No. A0208, Beyotime, China). Protein bands were detected using an Odyssey Infrared Imager system (LI-COR, NE, USA) and quantified with ImageJ software.

### Flow cytometry (FCM) assay

After 48 h of transfection, cells were stained with Annexin V-FITC (Cat. No. C1062M, Beyotime, China) and PI (Cat. No. C1008M, Beyotime, China), and apoptosis was analyzed by flow cytometry. The apoptosis rate was calculated by assessing early and late apoptotic cell populations.

### Statistical analysis

Statistical analyses were performed using GraphPad Prism (version 8.0.1) and R software (version 3.6.4). Data are presented as mean ± standard deviation (SD). For comparison of OAS3 expression between normal and tumor samples, unpaired Wilcoxon rank-sum test was used. The expression differences of the OAS3 across different clinical stages were analyzed using an unpaired Student’s *t*-test. For comparisons among multiple groups, one-way analysis of variance (ANOVA) followed by post hoc tests was applied. Spearman’s correlation was used to assess the relationship between OAS3 expression and other variables. Kaplan–Meier survival curves were generated to assess the correlation between OAS3 expression and patient survival. A *p*-value of < 0.05 was considered statistically significant.

## Results

### Differential expression of OAS3 across various tissues and cancer types

**Figure 1:**
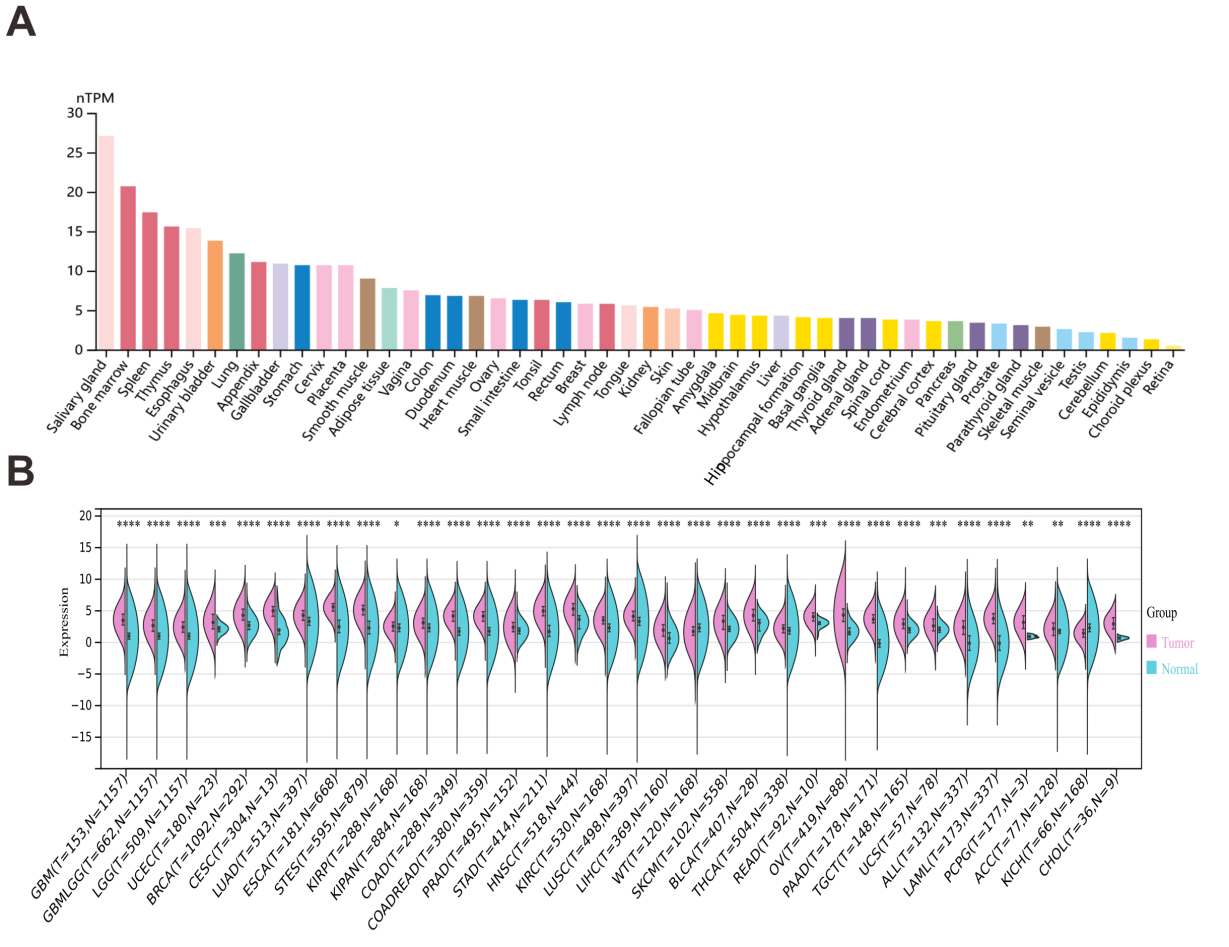
Analysis of OAS3 expression across human organs and tumor types. (A) OAS3 expression levels across various human organs. (B) OAS3 expression across 34 tumor types. *p < 0.05, **p < 0.01, ***p < 0.001, **** *p* < 0.001.

According to the HPA, OAS3 exhibits low tissue specificity, with cytoplasmic expression observed in most tissues. Notably, OAS3 is ubiquitously expressed across various human tissues, with particularly high expression in the salivary gland, bone marrow, and thymus, suggesting its involvement in fundamental cellular processes and the immune responses (Fig. 1A). Besides, OAS3 expression was significantly downregulated in Wilms tumor (WT) and kidney chromophobe (KICH), while it was upregulated in 32 other cancer types (Fig. 1B, Table S3). This suggests that OAS3 is globally upregulated in most malignancies relative to normal tissues, while its differential expression pattern across tumor types suggests context-dependent biological roles, acting as a putative oncogene in some cancers and a tumor suppressor in others.

### Stage-specific and cancer-specific expression patterns of OAS3 across multiple tumor types

**Figure 2:**
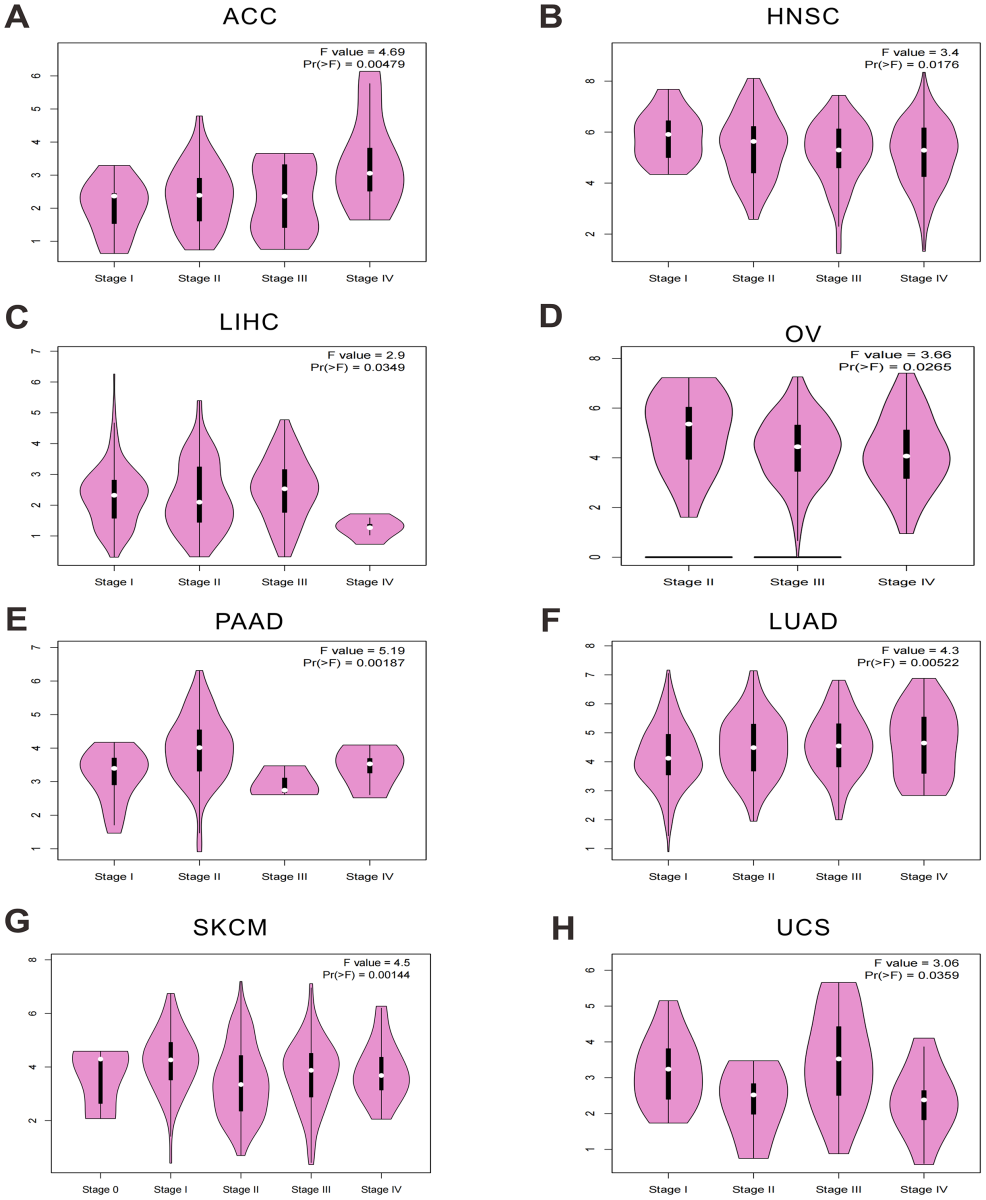
Stage-specific expression patterns of OAS3 across multiple cancer types. (A–H) OAS3 expression across different stages in ACC, HNSC, LIHC, OV, PAAD, LUAD, SKCM, UCS respectively.

In this study, OAS3 expression was analyzed across different stages of eight cancer types (adrenocortical carcinoma (ACC), head and neck squamous cell carcinoma (HNSC), liver hepatocellular carcinoma (LIHC), ovarian serous cystadenocarcinoma (OV), pancreatic adenocarcinoma (PAAD), lung adenocarcinoma (LUAD), skin cutaneous melanoma (SKCM), uterine carcinosarcoma (UCS)), revealing significant differences in its expression patterns (Figs. 2A–2H). In addition, OAS3 was found to exhibit cancer-specific expression patterns across different stages in LUAD, PAAD, pan-kidney cohort (KIPAN), HNSC, LIHC, mesothelioma (MESO), UCS, and ACC. In certain cancer types, OAS3 appeared to play a more critical role at either early or late stages, providing a foundation for further investigation into its functional roles in various tumor types (Fig. S1, Table S4). These findings suggest that the functions of OAS3 may be diverse across different cancers, with its activity closely associated with tumor staging and progression. Importantly, OAS3 was broadly upregulated in cancers overall and showed stage-dependent variation, with higher expression in advanced stages of several tumor types (ACC and LUAD).

### Prognostic significance of OAS3 expression in cancer survival outcomes

**Figure 3:**
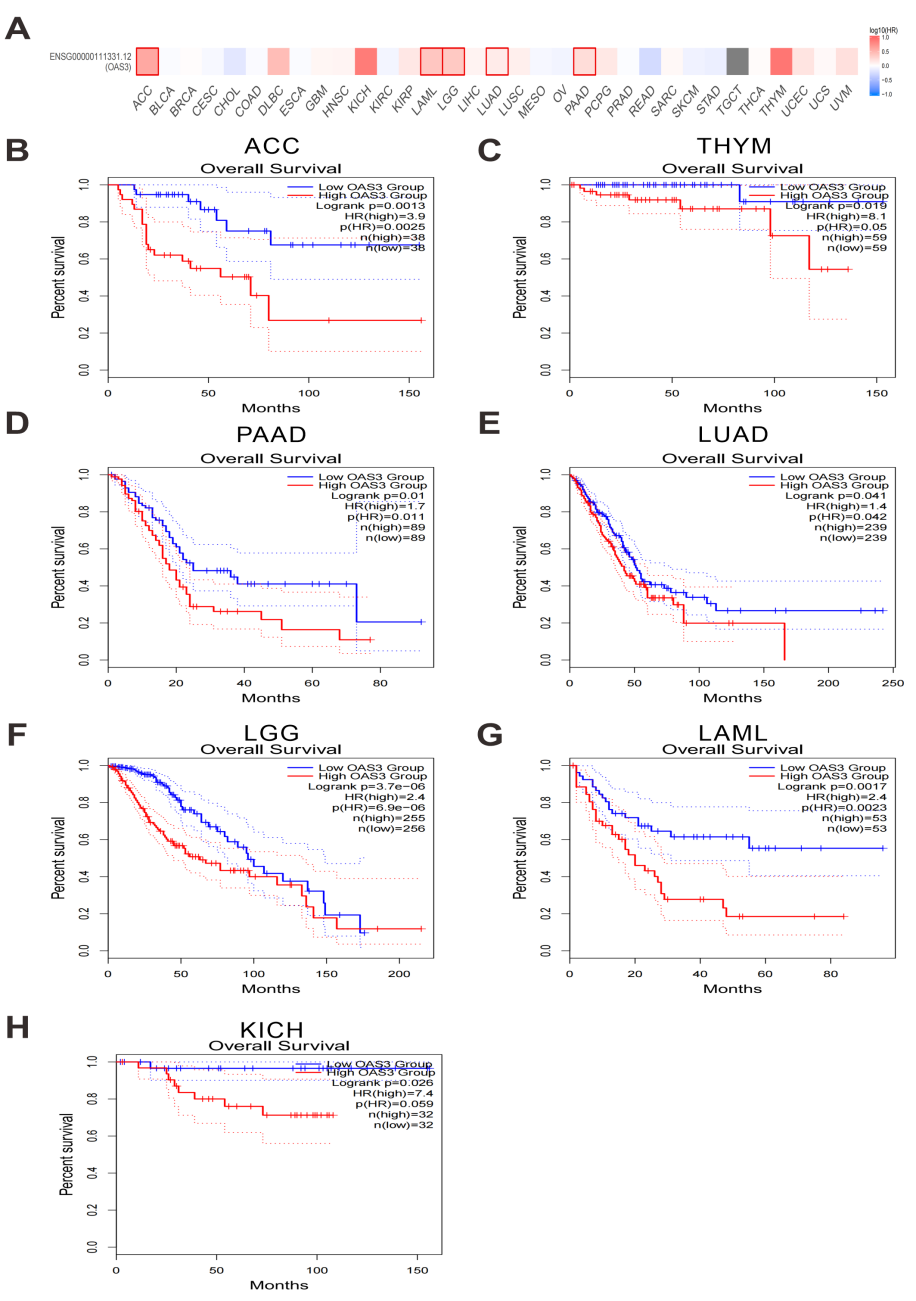
Kaplan–Meier survival curves of OAS3 expression on the OS across TCGA cancer types. Survival map of OAS3 expression on the OS (A–H) across various cancer types. The heatmap depicts the impact of OAS3 expression on the the OS across various cancer types (HR values). Red indicates that high OAS3 expression is associated with poorer OS, while blue indicates better OS.

High OAS3 expression was found to be significantly associated with poorer overall survival (OS) in several cancers, including ACC, thymoma (THYM), PAAD, LUAD, brain lower grade glioma (LGG), LAML, and KICH (Figs. 3A–3H). Additionally, it was correlated with shorter disease-free survival (DFS) in ACC, LGG, PAAD, and prostate adenocarcinoma (PRAD) (Figs. S2A–S2E). Based on these findings, its impact on both OS and DFS was highlighted, emphasizing its clinical relevance in cancer prognosis and as a potential therapeutic target. Moreover, Cox proportional hazards regression model analysis revealed that high OAS3 expression was significantly associated with poorer OS in several cancer types, particularly in glioblastoma & lower grade glioma (GBMLGG), LGG, LAML, KIPAN, ACC, PAAD, and LUAD, suggesting that OAS3 may serve as an adverse prognostic biomarker in these cancers. However, in certain cancers such as breast invasive carcinoma (BRCA), LIHC, and lung squamous cell carcinoma (LUSC), OAS3 expression showed no significant correlation with survival outcomes, indicating its limited prognostic relevance in these tumor types. Additionally, in a few cancers, including skin cutaneous melanoma (SKCM) and skin cutaneous melanoma - metastatic (SKCM-M), high OAS3 expression was associated with improved survival, suggesting a potential protective role in these cancers (Fig. S3).

### The dual role of OAS3 in modulating immune responses and tumor stemness

**Figure 4:**
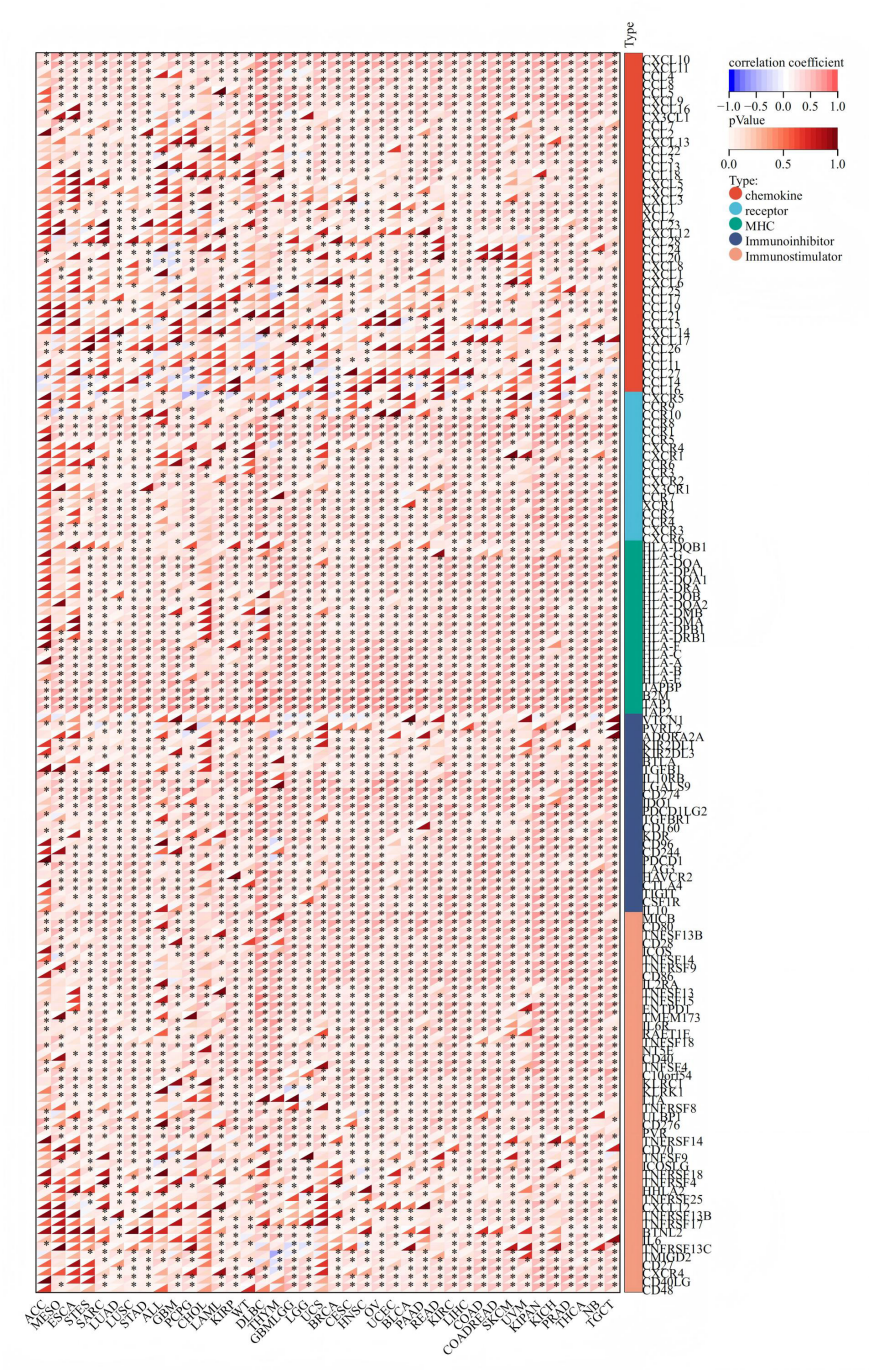
Correlation between OAS3 expression and immune-related genes.

**Figure 5:**
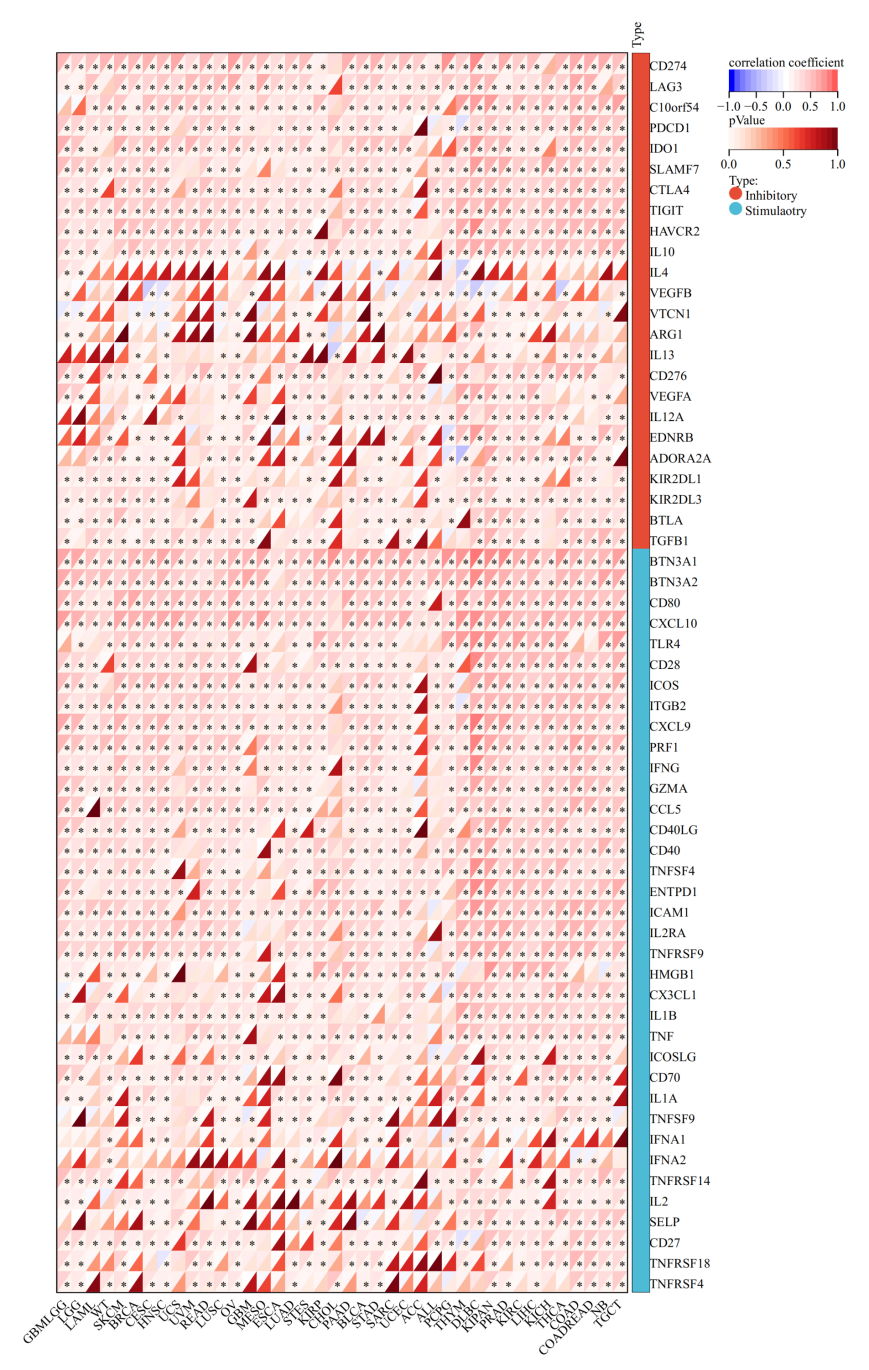
Correlation between OAS3 expression and immune checkpoints.

We explored the correlation between OAS3 expression and immune-related genes, OAS3 exhibited significant positive correlations with chemokines (*e.g.*, CCL and CXCL series) and receptors (*e.g.*, CCR and CXCR series), suggesting its role in immune cell recruitment and tumor microenvironment regulation. Additionally, its association with immunoinhibitors (*e.g.*, VTCN1, PVRL2) and immunostimulators (*e.g.*, CD86, CD80) implies a dual role in modulating immune responses (Fig. 4). In immune checkpoint analysis, OAS3 is significantly positively correlated with immune checkpoint molecules (*e.g.*, CTLA-4, CD274, LAG3) and immunostimulants (*e.g.*, CD80, CXCL10, CD28), suggesting that OAS3 may regulate the tumor immune microenvironment by enhancing immune suppression pathways and promoting immune responses (Fig. 5). Next, we revealed the complex relationship between OAS3 and tumor stemness, suggesting that OAS3 may influence tumor progression by regulating tumor stemness in certain cancer types (Fig. S4). A significant correlation was observed in 15 cancers, with a notable positive correlation in seven types (GBMLGG, LGG, PRAD, THYM, thyroid carcinoma (THCA), pheochromocytoma and paraganglioma (PCPG), and cholangiocarcinoma (CHOL)). In contrast, a significant negative correlation was found in 8 cancer types (colon adenocarcinoma (COAD), colon adenocarcinoma/rectum adenocarcinoma esophageal carcinoma (COADREAD), sarcoma (SARC), HNSC, LUSC, MESO, testicular germ cell tumors (TGCT), and bladder urothelial carcinoma (BLCA)). Notably, the strong correlation between OAS3 and tumor stemness in HNSC, KIPAN, and LGG suggests that OAS3 may play a crucial role in stemness maintenance and tumor progression in these cancers.

### Correlation between OAS3 expression and immune infiltration across cancers

**Figure 6:**
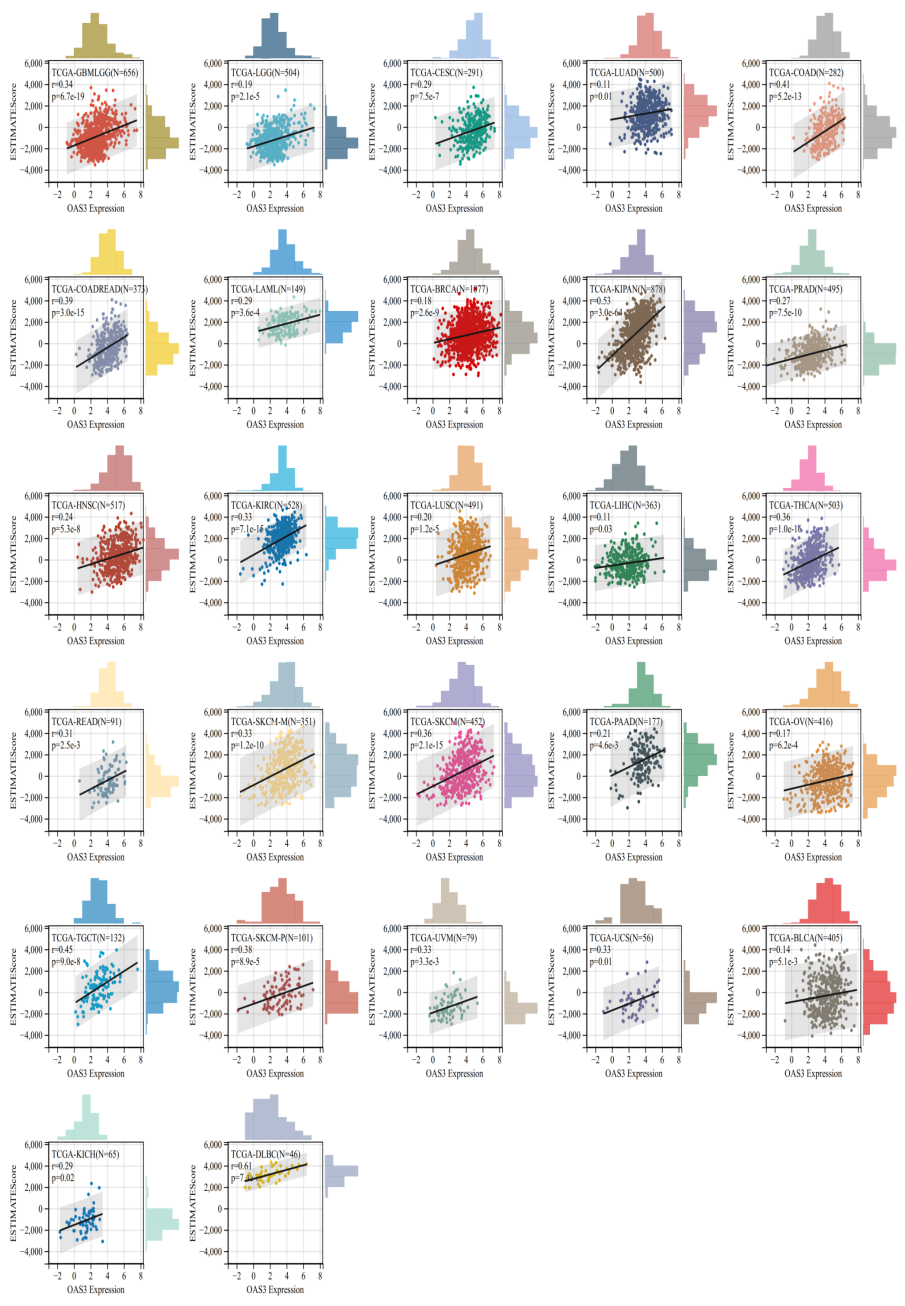
Correlation between OAS3 expression and tumor immune infiltration. Scatter plots and histograms presented the correlation between OAS3 expression and tumor immune infiltration across various cancer types. Each subplot represented a cancer type, with OAS3 expression on the *x*-axis and immune infiltration score on the *y*-axis.

OAS3 expression was significantly correlated with immune infiltration in 27 cancer types, all of which showed a positive correlation, including GBMLGG, LGG, BRCA, cervical squamous cell carcinoma and endocervical adenocarcinoma (CESC), LUAD, KIPAN, COAD, COADREAD, PRAD, HNSC, kidney renal clear cell carcinoma (KIRC), LUSC, LIHC, SKCM, BLCA, SKCM-M, THCA, rectum adenocarcinoma (READ), skin cutaneous melanoma - primary (SKCM-P), OV, uveal melanoma (UVM), PAAD, TGCT, UCS, LAML, lymphoid neoplasm diffuse large B-cell lymphoma (DLBC), and KICH (Fig. 6). This suggests that OAS3 may influence the immune response and tumor progression in these cancers by regulating the infiltration of immune cells. Besides, the analysis revealed significant correlations between OAS3 and various immune cell types, particularly with T regulatory cells (Tregs) and cancer-associated fibroblasts, suggesting that OAS3 may help shape an immunosuppressive microenvironment through interferon-related signaling that supports Treg activity and fibroblast activation. Additionally, significant correlations were observed with endothelial cells and hematopoietic stem cells, indicating its involvement in modulating immune cell dynamics within the tumor (Fig. S5). To further validate these findings, we applied both xCell and CIBERSORT algorithms. Consistently, OAS3 expression showed positive correlations with M1 macrophages across multiple cancers, while correlations with M2 macrophages were weak and heterogeneous. In addition, OAS3 was positively associated with Th2 cells in the xCell analysis (Fig. S6), a pattern indirectly supported by CD4^+^ T-cell subsets in CIBERSORT (Fig. S7). These results were obtained from unadjusted correlations, indicating reproducible cross-method patterns while not fully excluding the potential influence of immune admixture.

### Analysis of OAS3 interaction, correlation with immune genes, and functional pathways

**Figure 7:**
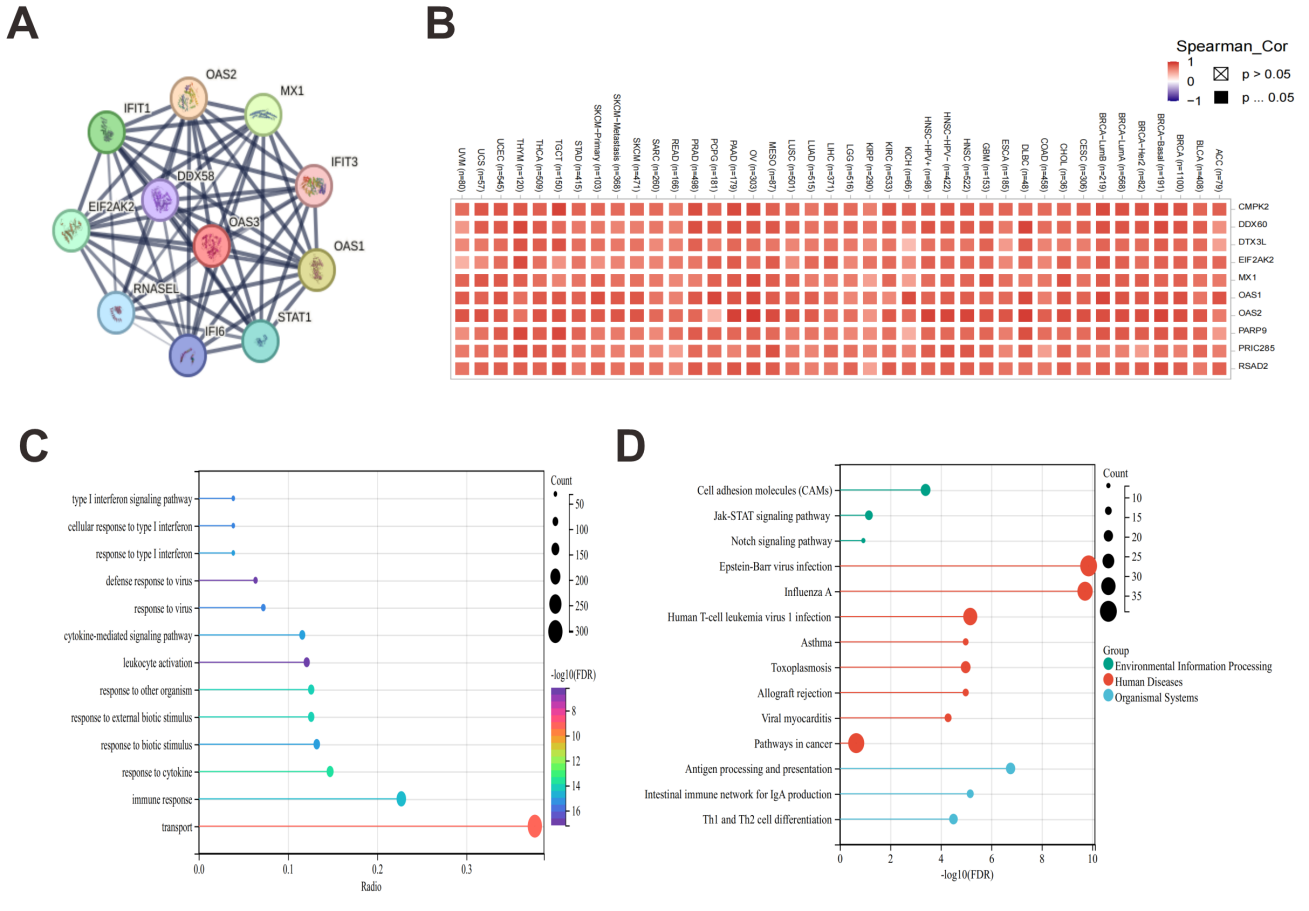
Protein interaction network, correlation, and enrichment analysis of genes associated with OAS3. (A) PPI network between OAS3 and its associated genes. Each node in the network represents a gene, and the connections between them illustrate their interactions. (B) Spearman correlation coefficients between OAS3 and several other genes across various cancer types from the TIMER2.0 Gene_Corr module. Each cell in the heatmap represents the correlation between OAS3 and a gene in a particular cancer type. The color intensity indicates the strength of the correlation, with darker red shades representing higher positive correlations, and lighter shades indicating weaker correlations. GO (C) and KEGG(D) enrichment analysis of genes associated with OAS3.

Based on STRING database, OAS3 was found to interact closely with several genes involved in antiviral responses, such as OAS1, OAS2, MX1, IFIT1, and IFIT3, suggesting that OAS3 may play a key role in interferon responses and immune regulation (Fig. 7A). After analyzing 100 genes related to OAS3 using the GEPIA2 tool, the top 10 genes were selected for further analysis using the Gene_Corr module on the strength of TIMER2.0. We observed that OAS3 is highly correlated with several immune-related genes, including MX1, OAS1, OAS2, and PARP9, across multiple cancer types (Fig. 7B). Then, GO enrichment analysis of genes associated with OAS3 revealed significant enrichment in biological processes, primarily focusing on immune responses and cytokine-mediated signaling pathways, such as the “type I interferon signaling pathway”, “cellular response to type I interferon”, “defense response” (Fig. 7C). Additionally, KEGG analysis revealed significant enrichment in pathways such as “cell adhesion molecules (CAMs)”, “Janus kinase-signal transducer and activator of transcription (JAK-STAT) signaling pathway”, and “Notch signaling pathway” (Fig. 7D).

### OAS3 mutation characteristics in the TCGA pan-cancer cohort

**Figure 8:**
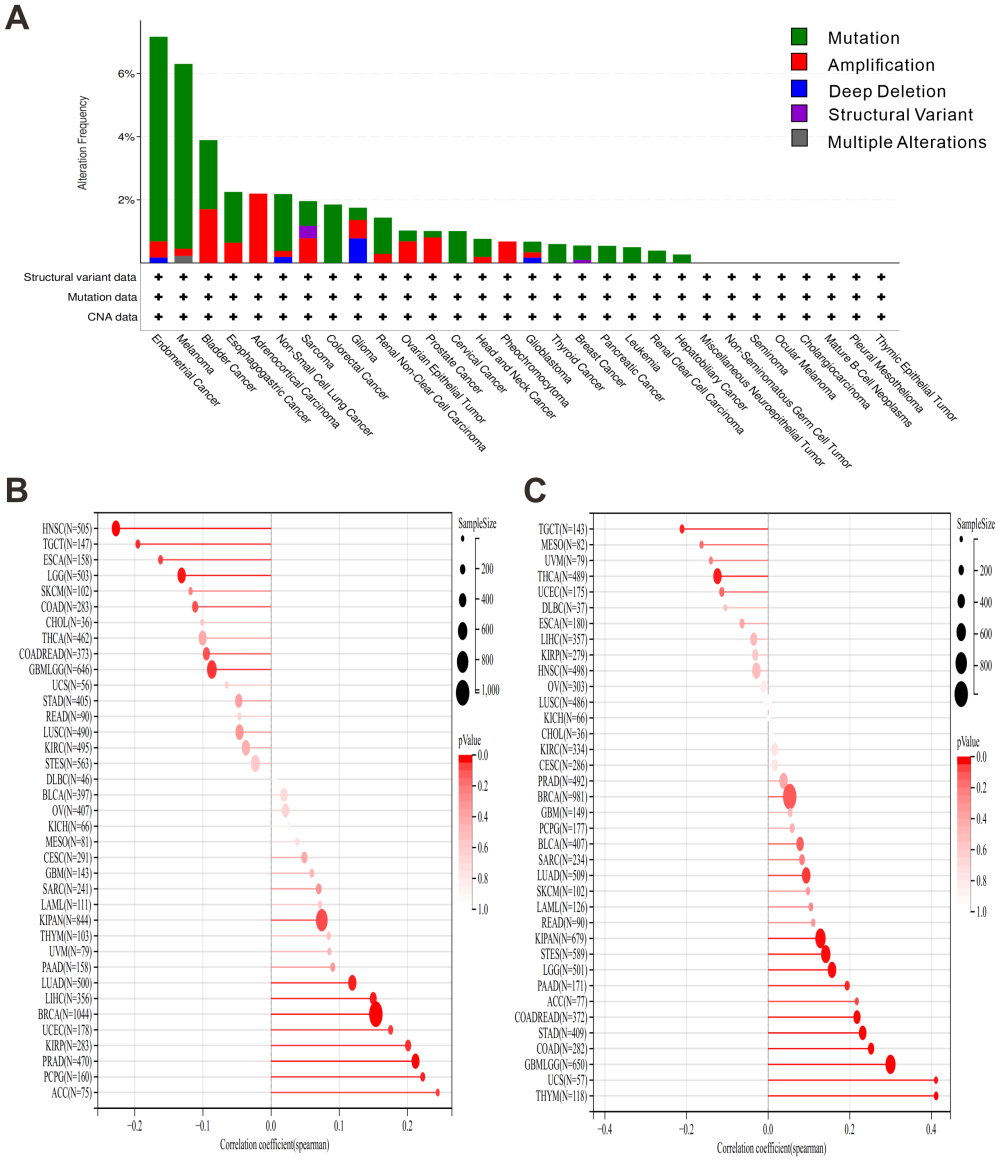
Analysis of associations of different genomic features associated with OAS3 expression. (A) The mutation analysis of OAS3 across different cancer types using the cBioPortal platform. The relationship between OAS3 gene and HRD (B), TMB (C).

To explore the frequency and types of OAS3 mutations in different tumors, mutation analysis using the cBioPortal platform was performed to investigate the relationship between these mutations and cancer development. OAS3 exhibited a relatively high mutation frequency in several cancer types, particularly in endometrial cancer and melanoma, with mutation frequencies exceeding 6% (Fig. 8A). In the HRD analysis, we observed that OAS3 was significantly correlated in 13 cancer types (Fig. 8B). Specifically, it exhibited a significantly positive correlation in eight cancer types (LUAD, BRCA, kidney renal papillary cell carcinoma (KIRP), PRAD, uterine corpus endometrial carcinoma (UCEC), LIHC, PCPG, ACC) and a significantly negative correlation in five cancer types (GBMLGG, LGG, esophageal carcinoma (ESCA), HNSC, TGCT). Additionally, TMB profiling demonstrated a positive correlation between OAS3 and TMB in several cancers, particularly in cancer types like GBMLGG, LGG, LUAD, COAD, COADREAD, stomach and esophageal carcinoma (STES), KIPAN, stomach adenocarcinoma (STAD), THYM, PAAD, and UCS, while a negative correlation was observed in THCA and TGCT (Fig. 8C). To further investigate whether OAS3 is involved in RNA modification processes and its potential role in regulating RNA modification-related genes in different cancers, we analyzed the correlation between OAS3 and genes involved in three types of RNA modifications (m1A, m5C, m6A). The analysis revealed significant correlations between OAS3 and several RNA modification-related genes, such as TRMT61A, YTHDF1, and ALKBH1 (Fig. S8).

### The function analysis of OAS3 in lung and breast cancer

**Figure 9:**
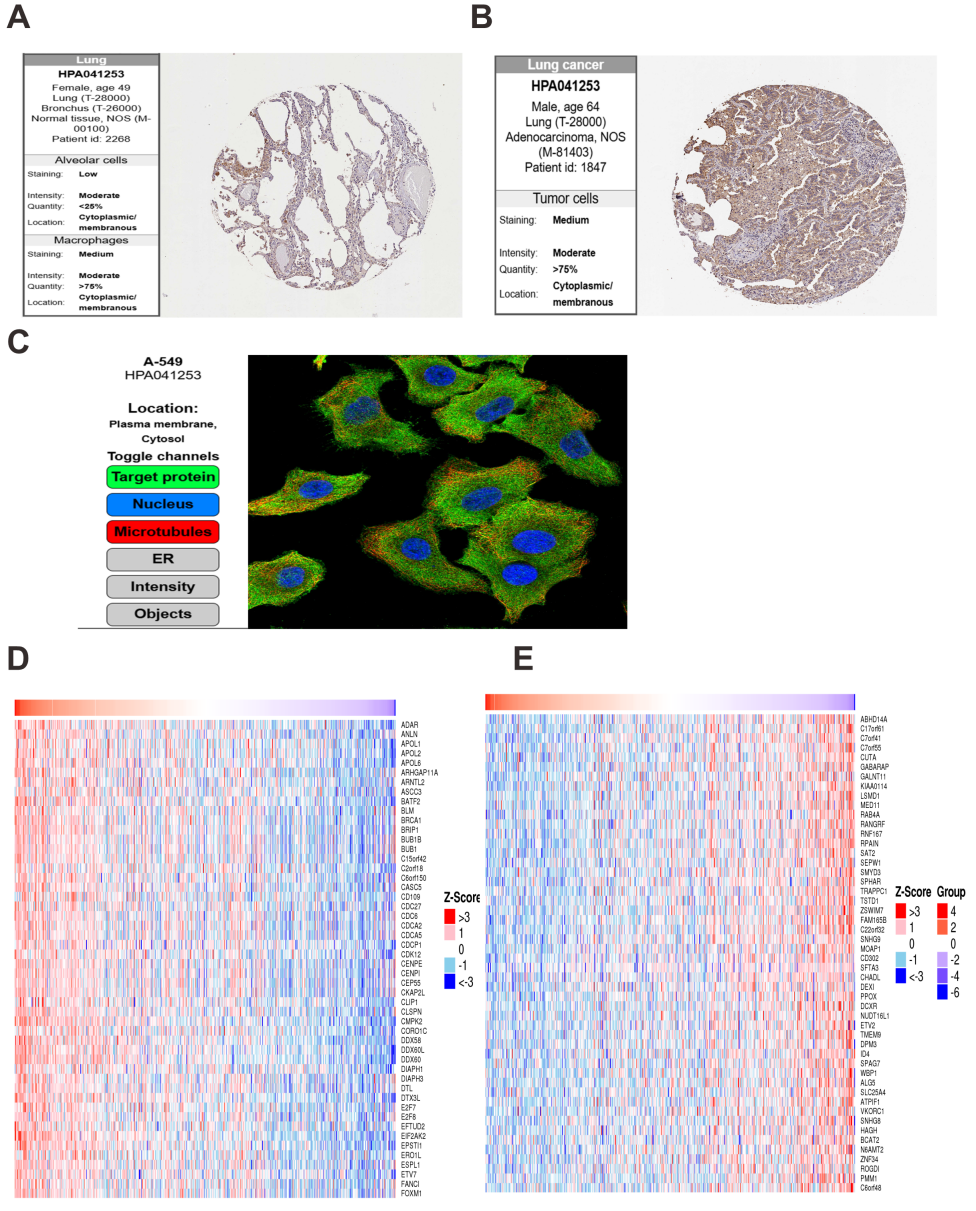
Analysis of OAS3 expression and correlations in lung cancer. (A) IHC results from HPA showed that OAS3 exhibited low staining intensity in alveolar cells. (B) OAS3 exhibited medium staining intensity in lung adenocarcinoma tumor cells. (C) Subcellular localization of OAS3 in A549 cells. The top 50 genes positively (D) and negatively (E) correlated with OAS3 in lung cancer were identified and visualized in a heatmap.

**Figure 10:**
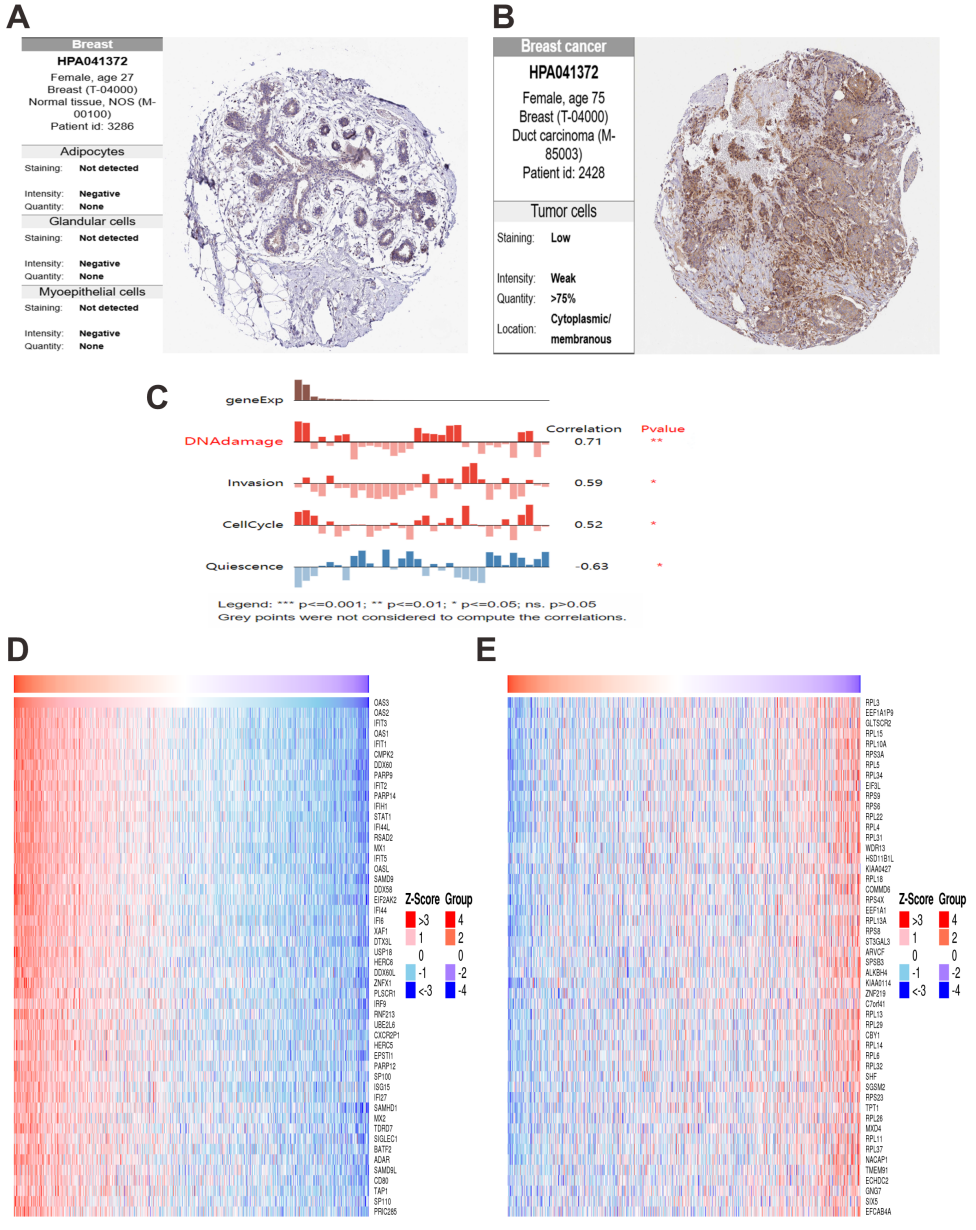
OAS3 expression and correlations in breast cancer. (A) OAS3 expression in normal breast tissue. (B) OAS3 expression in breast cancer tissue. (C) CancerSEA analysis of OAS3 function in breast cancer. Heatmaps show the top 50 genes positively (D) and negatively (E) correlated with OAS3.

In normal lung tissue, OAS3 was expressed at low levels in alveolar cells with weak staining and limited distribution (Fig. 9A). In contrast, lung cancer tissues showed significantly elevated OAS3 expression, with moderate staining in over 75% of tumor cells (Fig. 9B). IF analysis from the HPA database revealed OAS3 primarily localized in the cytosol and plasma membrane of A549 cells, indicated by green fluorescence, with nuclear (blue) and microtubule (red) staining as spatial references (Fig. 9C). LinkedOmics analysis identified the top 50 genes positively (Fig. 9D) and negatively (Fig. 9E) correlated with OAS3 in lung cancer. Positively correlated genes, such as BRCA1, BUB1, and CDC6, were linked to cell cycle regulation, suggesting OAS3’s role in promoting tumor proliferation. Negatively correlated genes, like MOAP1, DEXI, and RPAIN, suggested potential suppression by OAS3. KEGG pathway analysis highlighted enrichment in the “cell cycle”, “Epstein-Barr virus infection”, and “NOD-like receptor signaling pathway” (Fig. S9A). GO enrichment analysis revealed significant associations with processes like “mitotic cell cycle” and ”chromosome segregation”, indicating OAS3’s involvement in cell division and tumor proliferation (Fig. S9B). In normal breast tissue, OAS3 expression was absent in adipocytes, glandular cells, and myoepithelial cells (Fig. 10A). However, in breast cancer tissue, OAS3 was detected in over 75% of cells, with low expression and weak staining intensity (Fig. 10B). CancerSEA analysis showed that OAS3 in breast cancer cells was strongly positively correlated with DNA damage (0.71, *p* ≤ 0.01) and moderately with invasion (0.59, *p* ≤ 0.05) and the cell cycle (0.52, *p* ≤ 0.05). In contrast, OAS3 was negatively correlated with quiescence (−0.63, *p* ≤ 0.05) (Fig. 10C). Heatmaps revealed that positively correlated genes had high expression alongside OAS3 (Fig. 10D), while negatively correlated genes showed lower expression (Fig. 10E). These results suggest OAS3’s complex role in regulating processes like the cell cycle and immune responses in breast cancer.

### Survival curve analysis of OAS3 in LAML patients with different mutation types and function analysis

**Figure 11:**
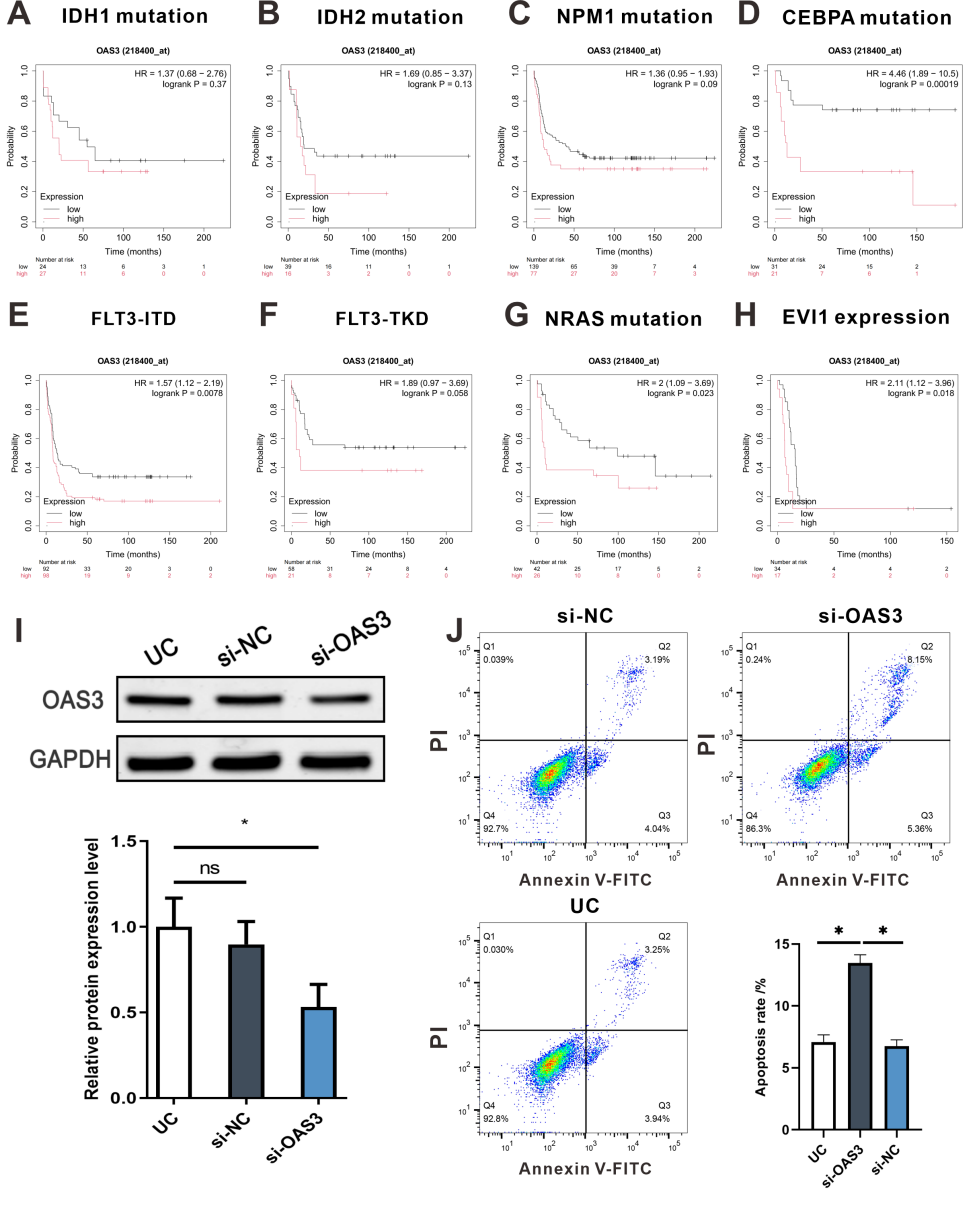
Survival curves of OAS3 expression in various mutations of LAML patients and vitro experiment. (A–H) IDH1, IDH2, NPM1, CEBPA, FLT3-ITD , FLT3-TKD, NRAS mutation, and EVI1 expression, with hazard ratios and log-rank *p*-values indicated. (I) Protein level of OAS3 in THP-1 after OAS3 knockdown (up). Statistical analysis(down) indicated that the relative OAS3 protein expression level in the si-OAS3 group was significantly lower than that in the si-NC/UG group, GAPDH was used as a loading control. (J) FCM was used to detect the apoptosis rate of OAS3 knockdown cells. *p < 0.05.

Kaplan–Meier survival analysis was performed to analysis the relationship between OAS3 expression and survival prognosis across various mutations in LAML patients. Interestingly, high OAS3 expression was associated with poor survival prognosis in patients with IDH1, IDH2, NPM1, CEBPA, FLT3, NRAS, and EVI1 mutations (Figs. 11A–11H). Notably, high OAS3 expression was significantly associated with lower survival in patients with CEBPA, FLT3-ITD, NRAS mutations, and EVI1 expression (*p* < 0.05). Next, we down-regulated the expression of OAS3 in leukemia cell THP-1 by transfection of interfering plasmids (Fig. 11I), and analyzed the apoptosis of cells after OAS3 knockdown by FCM. The results showed that the apoptosis rate was significantly increased in the si-OAS3 group (Fig. 11J). These results suggested that OAS3 may serve as a potential prognostic marker in LAML progression, closely linked to mutation types, and provide valuable insights for future clinical research.

## Discussion

Cancer biology complexity stems from interactions between tumor-intrinsic molecular changes and the dynamic tumor microenvironment, especially immune cell infiltration (Downs-Canner et al., 2022; Wu et al., 2025). The OAS3 gene, crucial in innate antiviral immunity, has gained attention for its diverse roles in various cancers across malignancies (Caglar et al., 2024; Deng et al., 2019; Li et al., 2022b; Zhang et al., 2024; Zhang & Yu, 2020). In lung adenocarcinoma (LUAD), OAS3, combined with other genes, helps predict patient survival and immunotherapy response (You & Shen, 2022). In non-small cell lung cancer (NSCLC), high OAS3 levels link to postoperative recurrence (Shi et al., 2022). Notably, elevated OAS3 expression correlated with postoperative recurrence in stage IB-IIIA NSCLC patients receiving neoadjuvant chemoimmunotherapy (Casarrubios et al., 2022). In gastric cancer, OAS3 is involved in drug resistance (Yu et al., 2018), while in breast cancer, it is a potential therapeutic target (Alam et al., 2022). Laryngeal squamous cell carcinoma (LSCC) analyses revealed OAS3’s prognostic value, with heightened expression correlating with poor survival through secretory/membrane-localized modulation of tumor-immune crosstalk (Yan et al., 2022). Pancreatic ductal adenocarcinoma (PDAC) studies identified OAS3 among 10 key genes influencing overall survival, suggesting its utility in microenvironment-targeted therapeutic strategies (Tang et al., 2019). In castration-resistant prostate cancer (CRPC), OAS3 overexpression in docetaxel-resistant PC-3R cell lines established its predictive potential for chemoresistance (Deng et al., 2019). Radiation response profiling demonstrated OAS3 co-upregulation with interferon-related genes (STAT1, G1P2, *etc.*) across irradiated breast, prostate, and glioma cell lines (Tsai et al., 2007). These results point to a context-dependent role of OAS3, which may influence tumor behavior and immune regulation in a cancer type specific manner.

Here, OAS3 is ubiquitously expressed across various human tissues. As demonstrated through TCGA pan-cancer dataset, we observed that OAS3 expression varies widely between different tumor types, and its levels are often associated with tumor grade and stage. Specifically, OAS3 was significantly upregulated in several cancers, including LUAD, BRCA, and LAML, aligning with the notion that OAS3 might be involved in tumor progression. In contrast, its expression in other cancers like WT and KICH showed a downregulated pattern, suggesting that OAS3 may have both tumor-suppressive and tumor-promoting roles, depending on the cancer type. Furthermore, our findings demonstrated that OAS3 dysregulation transcends simple overexpression or suppression, exhibiting stage-dependent modulation in eight cancer types (ACC, HNSC, LIHC, *etc.*) that correlates with disease progression trajectories. While OAS3 is generally upregulated in tumors compared with normal tissues, its expression also varies across clinical stages. This stage-dependent pattern suggests that OAS3 may be involved in cancer progression. Meanwhile, elevated OAS3 expression showed opposite prognostic trends across cancers, associated with poorer survival in some (*e.g.*, GBMLGG, LGG, LAML, KIPAN, ACC, PAAD, LUAD) but better outcomes in others (*e.g.*, SKCM, SKCM-M). These associations are exploratory and may reflect cancer-type–specific immune or epigenetic contexts rather than direct causal effects, warranting validation in multivariate and independent analyses. Immune landscape analysis is vital in cancer research, providing insights into immune cell distribution, function, and interactions within the tumor microenvironment. It aids in understanding immune evasion mechanisms, discovering new therapeutic targets, and supporting personalized treatment strategies (Guo et al., 2025; Lu et al., 2025). OAS3 showed co-expression with chemokine systems and immune checkpoints, indicating that it may influence both immune activation and suppression in a context-dependent manner. This immunological dichotomy is further evidenced by its divergent correlations with tumor-infiltrating lymphocytes—positive associations with Tregs and cancer-associated fibroblasts *versus* negative correlations with cytotoxic T cells in specific carcinomas. Although OAS3 expression showed predominantly positive correlations with immune infiltration across 27 cancers, several tumor types (*e.g.*, ACC, PCPG, WT, THYM, STES) displayed negative but non-significant trends. This indicates that the relationship between OAS3 and immune infiltration may vary by cancer type, reflecting biological heterogeneity rather than a universal effect. Such context-dependent associations highlight the importance of stratified analyses and warrant validation in larger, cancer-specific cohorts. This study reports unadjusted associations between OAS3 and immune infiltration. Consistent results across ESTIMATE, xCell, and CIBERSORT support the reliability of these patterns, mainly linked to M1 macrophages and variably to Th2 cells, though immune admixture and interferon (IFN) activity cannot be excluded. Future work should apply partial-correlation or regression approaches to better define tumor-intrinsic effects. Such findings align with emerging paradigms of interferon-stimulated genes (ISGs) functioning as double-edged swords in tumor immunity. Nevertheless, all correlations reported here were unadjusted, and immune admixture or IFN activity cannot be fully excluded. More rigorous approaches (*e.g.*, partial correlation or regression controlling for IFN scores and immune infiltration) will be needed to better define tumor-intrinsic effects. Besides, immune-related associations were assessed in a pooled pan-cancer manner. Stratified, cancer-specific analyses with meta-analysis would provide more precise insights, but were not feasible given current sample sizes and tools. Future work should incorporate such approaches when data availability allows.

Research indicates that the maintenance of cancer stemness depends on the precise regulation of key signaling pathways, including JAK/STAT, Hippo, and Notch. These pathways govern cancer stem cell self-renewal, proliferation, differentiation, and resistance to apoptosis, thereby promoting tumor growth and metastasis (Zeng et al., 2023). Additionally, their interactions collectively preserve the “stemness” of cancer stem cells, facilitating therapeutic evasion, drug resistance, and tumor progression (Chu et al., 2024). Notably, the stemness analysis reveals OAS3’s capacity to modulate cancer stem cell properties in a tumor-type-specific manner. The contrasting correlations observed in GBMLGG, LGG, PRAD (positive) *versus* COAD, COADREAD, SARC (negative), etc., may reflect differential activation of signaling pathways. OAS3 associated genes were enriched in classic stemness-related pathways such as JAK/STAT and Notch, consistent with well-established signaling programs (Chu et al., 2024; Zeng et al., 2023). These results should therefore be viewed as supportive evidence aligning OAS3 with known interferon- and stemness-regulating circuits, rather than novel discoveries (Ivashkiv & Donlin, 2014; Schneider, Chevillotte & Rice, 2014). The tumor-type–specific divergences observed in our analysis are therefore best regarded as preliminary, hypothesis-generating observations that require targeted experimental validation. In our study, stemness scores were derived from Sangerbox-standardized TCGA data, which reduces but does not completely remove purity-related bias, and thus these correlations should be interpreted cautiously and considered hypothesis-generating. Research has also shown that TRMT61A and YTHDF1 promote cancer cell growth, metastasis, and drug resistance by regulating RNA modification, translation, and stability. TRMT61A primarily affects protein synthesis by modulating tRNA methylation (Li, Zhang & Wang, 2022a), whereas YTHDF1 regulates RNA fate by recognizing m6A modifications (Jiang et al., 2021), thus playing a crucial role in tumor initiation and progression. In our study, correlations between OAS3 and RNA modification–related genes such as TRMT61A and YTHDF1 suggest a possible link between OAS3 expression and post-transcriptional regulatory processes. However, these associations are purely correlative, and further mechanistic studies are required to determine whether OAS3 directly participates in RNA modification or epitranscriptomic regulation. HRD and TMB are key cancer biomarkers, both are crucial for treatment response assessment and personalized therapy (Samstein et al., 2019; Toh & Ngeow, 2021). Correlation patterns between HRD and TMB suggest that OAS3 mutational status may be associated with genomic instability, potentially influencing responses to DNA damage–targeted therapies. However, these associations are correlative and exploratory, and further analyses controlling for confounding ISG expression and clinical factors are required to determine their predictive value.

Accumulation of DNA damage may lead to mutations in key genes that drive the development and progression of lung cancer (Sen, Gay & Byers, 2018; Venugopala, 2022). DNA damage, especially defects in BRCA1/BRCA2, impairs homologous recombination repair and contributes to the high incidence of breast cancer by promoting tumor initiation and progression (Voutsadakis & Digklia, 2023; Yamamoto & Hirasawa, 2021). This study highlighted the differential expression of OAS3 in lung and breast cancers, suggesting its role in tumor progression and DNA damage responses. In lung cancer, OAS3 was upregulated in over 75% of cells and correlated with cell cycle-related genes (*e.g.*, BRCA1, CDC6). Functional analyses implicated OAS3 in cell cycle regulation and immune response pathways. In breast cancer, although OAS3 expression was weaker, it was positively associated with DNA damage and invasion, and negatively with quiescence. However, these staining results remain correlative and cannot distinguish whether OAS3 originates from tumor or immune/stromal cells. They should be interpreted as preliminary findings consistent with transcriptomic data, pending confirmation by single-cell or spatial analyses to define OAS3’s cellular sources and functional roles. Consistent with previous studies reporting the adverse prognostic impact of FLT3-ITD, NRAS mutations, and EVI1 overexpression (Eshibona et al., 2022; Hinai & Valk, 2016; Liu et al., 2019), our analysis revealed that higher OAS3 expression correlated with shorter survival in these unfavorable LAML subsets. These observations are preliminary and correlative, and may reflect interactions between OAS3 and established adverse molecular features rather than an independent prognostic effect. Further functional or multivariate analyses will be necessary to clarify this relationship. In this study, LAML was selected for experimental validation because OAS3 is highly expressed and associated with poor prognosis in this malignancy. Our previous experiments in LAML cell lines demonstrated that OAS3 knockdown promotes apoptosis, inhibits proliferation, and induces cell-cycle arrest through the JAK-STAT signaling pathway (Zhang et al., 2025), consistent with the computational findings of this work.

Despite these findings, limitations warrant consideration. No batch-effect correction was applied, but consistent trends across datasets support the reliability of our findings. Future studies should apply dedicated batch-effect correction methods (*e.g.*, ComBat) to further refine these findings. Moreover, the lack of matched normal tissues in several TCGA cohorts limited our ability to evaluate baseline OAS3 expression differences between tumor and normal samples. As this study is based on bulk sequencing data, the observed OAS3 associations may reflect combined signals from tumor, immune, or stromal compartments. Single-cell or spatial transcriptomic validation will therefore be essential to determine whether OAS3 functions are tumor-intrinsic or immune-related. Likewise, the potential therapeutic relevance of OAS3 and its RNA modification–related interactions remain speculative and will require rigorous functional and *in vivo* validation before translational conclusions can be drawn.

## Conclusions

In summary, this study provides a comprehensive pan-cancer overview of OAS3, demonstrating its broad dysregulation, prognostic relevance, and consistent associations with immune-related features across diverse cancer types. These findings highlight OAS3 as a gene linked to tumor-associated immune and molecular characteristics in a context-dependent manner. However, the current findings remain descriptive and associative, and cannot determine whether OAS3 has a direct functional role in tumor development. Future work with mechanistic experiments and external validation cohorts is needed to establish its biological significance.

##  Supplemental Information

10.7717/peerj.20805/supp-1 Supplemental Information 1 Raw data of Figure 1 and Figures 3-8

10.7717/peerj.20805/supp-2 Supplemental Information 2 Supplemental tables

10.7717/peerj.20805/supp-3 Supplemental Information 3 Uncropped western blots for Figure 10I Protein level of OAS3 in THP-1 after OAS3 knockdown

10.7717/peerj.20805/supp-4 Supplemental Information 4 Comprehensive analysis of OAS3 expression across all TCGA cancer stages

10.7717/peerj.20805/supp-5 Supplemental Information 5 Kaplan-Meier survival curves of OAS3 expression on the DFS across various cancer types Survival map of OAS3 expression on the DFS (I-M) across various cancer types. The heatmap depicts the impact of OAS3 expression on the DFS across various cancer types (HR values). Red indicates that high OAS3 expression is associated with poorer DFS, while blue indicates better DFS.

10.7717/peerj.20805/supp-6 Supplemental Information 6 Cox proportional hazards regression analysis of OAS3 expression across TCGA cancer types Cox regression analysis of OAS3 expression across cancer types.

10.7717/peerj.20805/supp-7 Supplemental Information 7 Correlation between OAS3 expression and tumor stemness

10.7717/peerj.20805/supp-8 Supplemental Information 8 Correlation between OAS3 and a specific immune cell type, such as T cells, cancer-associated fibroblasts, endothelial cells, and hematopoietic stem cells Squares marked with an ” × ” represent p-values than 0.05.

10.7717/peerj.20805/supp-9 Supplemental Information 9 Correlation between OAS3 expression and immune cell infiltration across cancers based on the xCell algorithm

10.7717/peerj.20805/supp-10 Supplemental Information 10 CIBERSORT-based analysis of the relationship between OAS3 expression and immune cell composition in pan-cancer datasets

10.7717/peerj.20805/supp-11 Supplemental Information 11 Analysis of associations of RNA modification genes associated with OAS3 expression

10.7717/peerj.20805/supp-12 Supplemental Information 12 Analysis of OAS3-related pathway enrichments in lung cancer KEGG (A) and GO(B) pathway enrichment analysis of genes correlated with OAS3 in lung cancer.

## Additional Information and Declarations

### Data Availability

The following information was supplied regarding data availability: The data is available in the Supplemental Files. The Flow Cytometry Data are available at Zenodo: Zhang, X. (2025). FCM was used to detect the apoptosis rate of OAS3 knockdown cells. Zenodo. https://doi.org/10.5281/zenodo.17431965.
