# Supplementary material for: Pan-cancer multi-omics profiling of OAS3 reveals its immunological and prognostic associations across human cancers
Source: PeerJ. 2026 Feb 12;14:e20805. doi: 10.7717/peerj.20805 (PMC12906706; doi:10.7717/peerj.20805)
Supplement: Supplemental Information 2 [file peerj-14-20805-s002.pdf]

Table S1. Abbreviations of the cancer types included in the study

Cancer type abbreviation	Full name of cancer type
TCGA-ACC	Adrenocortical carcinoma
TCGA-BLCA	Bladder Urothelial Carcinoma
TCGA-BRCA	Breast invasive carcinoma
TCGA-CESC	Cervical squamous cell carcinoma and endocervical adenocarcinoma
TCGA-CHOL	Cholangiocarcinoma
TCGA-COAD	Colon adenocarcinoma
TCGA-COADREAD	Colon adenocarcinoma/Rectum adenocarcinoma Esophageal carcinoma
TCGA-ESCA	Esophageal carcinoma
TCGA-GBM	Glioblastoma multiforme
TCGA-GBMLGG	Glioma
TCGA-HNSC	Head and Neck squamous cell carcinoma
TCGA-KICH	Kidney Chromophobe
TCGA-KIPAN	Pan-kidney cohort (KICH+KIRC+KIRP)
TCGA-KIRC	Kidney renal clear cell carcinoma
TCGA-KIRP	Kidney renal papillary cell carcinoma
TCGA-LAML	Acute Myeloid Leukemia
TCGA-LGG	Brain Lower Grade Glioma
TCGA-LIHC	Liver hepatocellular carcinoma
TCGA-LUAD	Lung adenocarcinoma
TCGA-LUSC	Lung squamous cell carcinoma
TCGA-MESO	Mesothelioma
TCGA-OV	Ovarian serous cystadenocarcinoma
TCGA-PAAD	Pancreatic adenocarcinoma
TCGA-PCPG	Pheochromocytoma and Paraganglioma
TCGA-PRAD	Prostate adenocarcinoma
TCGA-READ	Rectum adenocarcinoma
TCGA-SARC	Sarcoma
TCGA-STAD	Stomach adenocarcinoma
TCGA-SKCM	Skin Cutaneous Melanoma
TCGA-STES	Stomach and Esophageal carcinoma
TCGA-TGCT	Testicular Germ Cell Tumors
TCGA-THCA	Thyroid carcinoma
TCGA-THYM	Thymoma

TCGA-UCEC	Uterine Corpus Endometrial Carcinoma
TCGA-UCS	Uterine Carcinosarcoma
TCGA-UVM	Uveal Melanoma
TARGET-OS	Osteosarcoma
TARGET-ALL	Acute Lymphoblastic Leukemia
TARGET-NB	Neuroblastoma
TARGET-WT	High-Risk Wilms Tumor

Table S2. The top 100 genes associated with OAS3 expression

Gene Symbol	Gene ID	PCC
OAS1	ENSG00000089127.12	0.8
CMPK2	ENSG00000134326.11	0.79
EIF2AK2	ENSG00000055332.16	0.77
OAS2	ENSG00000111335.12	0.76
HELZ2	ENSG00000130589.16	0.75
MX1	ENSG00000157601.13	0.74
DTX3L	ENSG00000163840.9	0.73
PARP9	ENSG00000138496.16	0.7
RSAD2	ENSG00000134321.11	0.7
DDX60	ENSG00000137628.16	0.7
IFIT3	ENSG00000119917.13	0.7
DDX58	ENSG00000107201.9	0.69
STAT1	ENSG00000115415.18	0.69
USP18	ENSG00000184979.9	0.67
UBE2L6	ENSG00000156587.15	0.66
EPSTI1	ENSG00000133106.14	0.65
IFI44L	ENSG00000137959.15	0.63
ZNFX1	ENSG00000124201.14	0.63
PARP14	ENSG00000173193.13	0.62
PLSCR1	ENSG00000188313.12	0.62
PARP12	ENSG00000059378.12	0.61
SAMD9L	ENSG00000177409.11	0.6
OASL	ENSG00000135114.12	0.6
ADAR	ENSG00000160710.15	0.59
NMI	ENSG00000123609.10	0.59
IFIT1	ENSG00000185745.9	0.58
TAP1	ENSG00000168394.10	0.58
TRIM14	ENSG00000106785.14	0.57
IFI35	ENSG00000068079.7	0.56
APOL6	ENSG00000221963.5	0.56
SP110	ENSG00000135899.16	0.56
SAMD9	ENSG00000205413.7	0.56
IFI27	ENSG00000165949.12	0.55
TAP2	ENSG00000204267.13	0.55
TRIM21	ENSG00000132109.9	0.53
IFIT5	ENSG00000152778.8	0.53
PML	ENSG00000140464.19	0.53
LAP3	ENSG00000002549.12	0.52
ETV7	ENSG00000010030.13	0.52
RTP4	ENSG00000136514.2	0.52
ISG15	ENSG00000187608.8	0.52
IFI6	ENSG00000126709.14	0.52
IFIT2	ENSG00000119922.8	0.52
RNF213	ENSG00000173821.19	0.51
PATL1	ENSG00000166889.13	0.51
MB21D1	ENSG00000164430.15	0.51
IRF9	ENSG00000213928.8	0.5
SLFN5	ENSG00000166750.9	0.5
BAK1	ENSG00000030110.12	0.49
MX2	ENSG00000183486.12	0.49
HCP5	ENSG00000206337.10	0.49
BATF2	ENSG00000168062.9	0.49
PTBP3	ENSG00000119314.15	0.49
NT5C3A	ENSG00000122643.18	0.49
MOB1A	ENSG00000114978.17	0.49
CEP55	ENSG00000138180.15	0.48
MKI67	ENSG00000148773.12	0.48

TUBA1C	ENSG00000167553.14	0.48
FBXO6	ENSG00000116663.10	0.48
G3BP1	ENSG00000145907.14	0.48
LSM12	ENSG00000161654.9	0.47
HERC6	ENSG00000138642.14	0.47
AC009950.1	ENSG00000280755.1	0.47
TAPBP	ENSG00000231925.11	0.47
FAM120A	ENSG00000048828.16	0.47
IFIH1	ENSG00000115267.5	0.47
ZDHHC5	ENSG00000156599.10	0.46
MELK	ENSG00000165304.7	0.46
XAF1	ENSG00000132530.16	0.46
ZC3HAV1	ENSG00000105939.12	0.46
IFITM1	ENSG00000185885.15	0.46
LGALS3BP	ENSG00000108679.12	0.46
KIF20B	ENSG00000138182.14	0.46
YWHAZ	ENSG00000164924.17	0.46
FAM83H	ENSG00000180921.6	0.46
MASTL	ENSG00000120539.14	0.46
PSMB9	ENSG00000240065.7	0.46
UBA6	ENSG00000033178.12	0.45
RRM2	ENSG00000171848.13	0.45
ECT2	ENSG00000114346.13	0.45
CDCP1	ENSG00000163814.7	0.45
TRIM26	ENSG00000234127.8	0.45
PSME3	ENSG00000131467.10	0.45
PPP2R5E	ENSG00000154001.13	0.45
TYMP	ENSG00000025708.12	0.45
PSMB2	ENSG00000126067.11	0.45
NAA50	ENSG00000121579.12	0.45
KIF11	ENSG00000138160.5	0.45
CKAP2L	ENSG00000169607.12	0.45
CPSF2	ENSG00000165934.12	0.45
PRPF40A	ENSG00000196504.15	0.45
RPS6KA4	ENSG00000162302.12	0.45
ACTR3	ENSG00000115091.11	0.45
RACGAP1	ENSG00000161800.12	0.45
PSMB8	ENSG00000204264.8	0.45
GTF2H3	ENSG00000111358.13	0.44
ELF4	ENSG00000102034.16	0.44
PGAM5	ENSG00000247077.6	0.44
BST2	ENSG00000130303.12	0.44
RAB8A	ENSG00000167461.11	0.44

Table S3. Differential expression of OAS3 across various cancer types and normal control

Cancer Type	Tumor expression level (mean ± SD)	Normal expression level (mean ± SD)	*P* value
GBM	3.60±1.34	0.97±1.26	1.80E-72
GBMLGG	2.75±1.40	0.97±1.26	3.30E-150
LGG	2.50±1.32	0.97±1.26	1.10E-110
UCEC	3.26±1.59	2.12±0.64	1.80E-04
BRCA	4.37±1.34	2.69±0.97	2.10E-76
CESC	4.84±1.20	1.78±1.71	4.00E-07
LUAD	4.26±1.16	3.29±1.12	5.10E-38
ESCA	5.56±1.02	2.46±1.54	2.40E-85
STES	5.11±1.16	2.28±1.61	2.70E-184
KIRP	2.49±1.05	2.20±1.43	0.02
KIPAN	2.96±1.19	2.20±1.43	8.70E-15
COAD	4.14±1.06	1.67±1.58	1.10E-83
COADREAD	4.12±1.04	1.71±1.57	4.10E-96
PRAD	2.38±1.31	1.86±0.90	2.70E-08
STAD	4.91±1.17	1.73±1.71	5.60E-77
HNSC	5.18±1.33	3.21±1.39	1.10E-14
KIRC	3.42±0.99	2.20±1.43	9.60E-34
LUSC	4.14±1.08	3.29±1.12	2.50E-31
LIHC	1.88±1.38	0.80±1.35	1.20E-17
SKCM	3.22±1.59	2.14±0.73	4.00E-14
BLCA	4.28±1.33	2.85±1.21	5.10E-07
THCA	2.19±1.06	1.88±1.11	1.70E-06
READ	4.05±0.99	2.95±0.57	3.40E-04
OV	4.20±1.65	1.75±0.89	8.00E-35
PAAD	3.68±1.17	-0.12±1.54	1.70E-52
TGCT	2.95±1.25	1.96±0.60	3.20E-16
UCS	2.78±1.44	2.04±0.72	1.70E-04
ALL	2.40±1.54	-0.08±1.91	4.60E-32
LAML	3.78±1.29	-0.08±1.91	3.90E-63
PCPG	3.13±1.43	0.94±0.26	9.70E-03
ACC	2.13±1.43	1.60±1.29	3.20E-03
CHOL	2.91±1.31	0.68±0.22	6.30E-05
WT	1.67±1.63	2.20±1.43	5.00E-05
KICH	1.33±1.05	2.20±1.43	2.00E-09

Table S4.The expression differences of OAS3 in different clinical stage samples within each tumor

Label	Comparison group(Mean±std)	Control group(Mean±std)	*t* test	Analysis of Variance(*ANOVA*)
CESC(Stage I=162,II=69,III=45,IV=21)	Stage II(4.94±1.25)	Stage I(4.79±1.11)	0.41	0.81
	Stage II(4.94±1.25)	Stage III(4.91±1.35)	0.93	
	Stage II(4.94±1.25)	Stage IV(4.92±1.25)	0.96	
	Stage I(4.79±1.11)	Stage III(4.91±1.35)	0.58	
	Stage I(4.79±1.11)	Stage IV(4.92±1.25)	0.66	
	Stage III(4.91±1.35)	Stage IV(4.92±1.25)	0.98	
LUAD(Stage I=274,II=122,III=83,IV=26)	Stage I(4.11±1.15)	Stage III(4.53±1.09)	0.003	**0.0043**
	Stage I(4.11±1.15)	Stage II(4.41±1.14)	0.01	
	Stage I(4.11±1.15)	Stage IV(4.56±1.25)	0.08	
	Stage III(4.53±1.09)	Stage II(4.41±1.14)	0.49	
	Stage III(4.53±1.09)	Stage IV(4.56±1.25)	0.89	
	Stage II(4.41±1.14)	Stage IV(4.56±1.25)	0.58	
COAD(Stage I=44,II=110,III=82,IV=40)	Stage II(4.11±1.12)	Stage III(4.18±1.01)	0.66	0.63
	Stage II(4.11±1.12)	Stage IV(3.94±1.12)	0.41	
	Stage II(4.11±1.12)	Stage I(4.22±1.01)	0.58	
	Stage III(4.18±1.01)	Stage IV(3.94±1.12)	0.25	
	Stage III(4.18±1.01)	Stage I(4.22±1.01)	0.86	
	Stage IV(3.94±1.12)	Stage I(4.22±1.01)	0.24	
COADREAD(Stage I=56,II=134,III=115,IV=53)	Stage II(4.14±1.09)	Stage III(4.11±1.06)	0.79	0.42
	Stage II(4.14±1.09)	Stage IV(3.90±1.07)	0.16	
	Stage II(4.14±1.09)	Stage I(4.21±0.96)	0.66	
	Stage III(4.11±1.06)	Stage IV(3.90±1.07)	0.23	
	Stage III(4.11±1.06)	Stage I(4.21±0.96)	0.51	
	Stage IV(3.90±1.07)	Stage I(4.21±0.96)	0.11	
BRCA(Stage I=182,II=617,III=248,IV=20)	Stage III(4.43±1.41)	Stage II(4.38±1.33)	0.64	0.41
	Stage III(4.43±1.41)	Stage I(4.24±1.28)	0.14	
	Stage III(4.43±1.41)	Stage IV(4.13±1.34)	0.35	
	Stage II(4.38±1.33)	Stage I(4.24±1.28)	0.19	
	Stage II(4.38±1.33)	Stage IV(4.13±1.34)	0.42	
	Stage I(4.24±1.28)	Stage IV(4.13±1.34)	0.73	
ESCA(Stage I=18,II=80,III=61,IV=16)	Stage II(5.69±1.01)	Stage I(5.43±1.04)	0.36	0.55
	Stage II(5.69±1.01)	Stage III(5.49±1.00)	0.25	
	Stage II(5.69±1.01)	Stage IV(5.42±1.05)	0.36	
	Stage I(5.43±1.04)	Stage III(5.49±1.00)	0.84	
	Stage I(5.43±1.04)	Stage IV(5.42±1.05)	0.97	
	Stage III(5.49±1.00)	Stage IV(5.42±1.05)	0.81	
STES(Stage I=76,II=201,III=230,IV=57)	Stage II(5.22±1.13)	Stage I(5.01±1.33)	0.23	0.4
	Stage II(5.22±1.13)	Stage III(5.08±1.16)	0.19	
	Stage II(5.22±1.13)	Stage IV(5.03±0.91)	0.18	
	Stage I(5.01±1.33)	Stage III(5.08±1.16)	0.71	
	Stage I(5.01±1.33)	Stage IV(5.03±0.91)	0.94	
	Stage III(5.08±1.16)	Stage IV(5.03±0.91)	0.73	
KIRP(Stage I=177,II=25,III=52,IV=16)	Stage I(2.44±1.06)	Stage III(2.62±1.10)	0.3	0.45
	Stage I(2.44±1.06)	Stage II(2.76±0.94)	0.13	
	Stage I(2.44±1.06)	Stage IV(2.47±1.07)	0.91	
	Stage III(2.62±1.10)	Stage II(2.76±0.94)	0.58	
	Stage III(2.62±1.10)	Stage IV(2.47±1.07)	0.64	
	Stage II(2.76±0.94)	Stage IV(2.47±1.07)	0.4	
KIPAN(Stage I=464,II=107,III=189,IV=103)	Stage I(2.96±1.16)	Stage III(3.03±1.20)	0.53	**0.00094**
	Stage I(2.96±1.16)	Stage II(2.63±1.34)	0.02	
	Stage I(2.96±1.16)	Stage IV(3.28±1.10)	0.0097	
	Stage III(3.03±1.20)	Stage II(2.63±1.34)	0.01	
	Stage III(3.03±1.20)	Stage IV(3.28±1.10)	0.07	
	Stage II(2.63±1.34)	Stage IV(3.28±1.10)	0.00015	
STAD(Stage I=58,II=121,III=169,IV=41)	Stage II(4.92±1.11)	Stage III(4.93±1.17)	0.93	0.99
	Stage II(4.92±1.11)	Stage I(4.88±1.39)	0.87	
	Stage II(4.92±1.11)	Stage IV(4.88±0.81)	0.8	
	Stage III(4.93±1.17)	Stage I(4.88±1.39)	0.82	
	Stage III(4.93±1.17)	Stage IV(4.88±0.81)	0.73	
	Stage I(4.88±1.39)	Stage IV(4.88±0.81)	0.98	
UCEC(Stage I=98,II=24,III=48,IV=10)	Stage I(3.21±1.52)	Stage II(3.18±1.63)	0.94	0.86
	Stage I(3.21±1.52)	Stage III(3.32±1.72)	0.7	
	Stage I(3.21±1.52)	Stage IV(3.62±1.68)	0.47	
	Stage II(3.18±1.63)	Stage III(3.32±1.72)	0.74	
	Stage II(3.18±1.63)	Stage IV(3.62±1.68)	0.49	
	Stage III(3.32±1.72)	Stage IV(3.62±1.68)	0.61	
HNSC(Stage I=27,II=82,III=93,IV=316)	Stage IV(5.09±1.34)	Stage I(5.81±0.97)	0.001	**0.02**
	Stage IV(5.09±1.34)	Stage II(5.39±1.31)	0.07	
	Stage IV(5.09±1.34)	Stage III(5.11±1.33)	0.89	
	Stage I(5.81±0.97)	Stage II(5.39±1.31)	0.08	
	Stage I(5.81±0.97)	Stage III(5.11±1.33)	0.004	
	Stage II(5.39±1.31)	Stage III(5.11±1.33)	0.17	
KIRC(Stage I=266,II=57,III=123,IV=81)	Stage III(3.41±0.99)	Stage II(3.17±1.13)	0.18	0.15
	Stage III(3.41±0.99)	Stage IV(3.57±0.92)	0.24	
	Stage III(3.41±0.99)	Stage I(3.43±0.98)	0.84	
	Stage II(3.17±1.13)	Stage IV(3.57±0.92)	0.03	
	Stage II(3.17±1.13)	Stage I(3.43±0.98)	0.12	
	Stage IV(3.57±0.92)	Stage I(3.43±0.98)	0.24	
LUSC(Stage I=242,II=161,III=84,IV=7)	Stage I(4.09±1.10)	Stage III(4.00±1.12)	0.52	0.08
	Stage I(4.09±1.10)	Stage II(4.30±1.02)	0.04	
	Stage I(4.09±1.10)	Stage IV(3.71±0.89)	0.31	
	Stage III(4.00±1.12)	Stage II(4.30±1.02)	0.04	
	Stage III(4.00±1.12)	Stage IV(3.71±0.89)	0.45	
	Stage II(4.30±1.02)	Stage IV(3.71±0.89)	0.13	
THYM(Stage I=36,II=61,III=14,IV=6)	Stage I(2.84±1.17)	Stage III(3.10±1.51)	0.57	0.86
	Stage I(2.84±1.17)	Stage II(2.79±1.47)	0.83	
	Stage I(2.84±1.17)	Stage IV(3.09±1.54)	0.72	
	Stage III(3.10±1.51)	Stage II(2.79±1.47)	0.49	
	Stage III(3.10±1.51)	Stage IV(3.09±1.54)	0.99	
	Stage II(2.79±1.47)	Stage IV(3.09±1.54)	0.66	
LIHC(Stage I=169,II=86,III=85,IV=5)	Stage I(1.83±1.29)	Stage II(1.79±1.52)	0.85	**0.05**
	Stage I(1.83±1.29)	Stage III(2.09±1.39)	0.15	
	Stage I(1.83±1.29)	Stage IV(0.44±0.65)	0.0066	
	Stage II(1.79±1.52)	Stage III(2.09±1.39)	0.18	
	Stage II(1.79±1.52)	Stage IV(0.44±0.65)	0.0052	
	Stage III(2.09±1.39)	Stage IV(0.44±0.65)	0.002	
THCA(Stage I=283,II=52,III=112,IV=55)	Stage I(2.27±1.01)	Stage III(2.13±1.13)	0.25	0.22
	Stage I(2.27±1.01)	Stage II(1.97±1.08)	0.06	
	Stage I(2.27±1.01)	Stage IV(2.16±1.10)	0.49	
	Stage III(2.13±1.13)	Stage II(1.97±1.08)	0.38	
	Stage III(2.13±1.13)	Stage IV(2.16±1.10)	0.87	
	Stage II(1.97±1.08)	Stage IV(2.16±1.10)	0.36	
MESO(Stage I=10,II=16,III=45,IV=16)	Stage I(4.84±1.12)	Stage II(3.87±1.28)	0.05	
	Stage I(4.84±1.12)	Stage III(3.80±1.25)	0.02	

	Stage I(4.84±1.12)	Stage IV(4.47±1.20)	0.43	**0.05**
	Stage II(3.87±1.28)	Stage III(3.80±1.25)	0.85	
	Stage II(3.87±1.28)	Stage IV(4.47±1.20)	0.18	
	Stage III(3.80±1.25)	Stage IV(4.47±1.20)	0.07	
READ(Stage I=12,II=24,III=33,IV=13)	Stage III(3.92±1.18)	Stage I(4.20±0.82)	0.37	
	Stage III(3.92±1.18)	Stage II(4.28±0.93)	0.2	
	Stage III(3.92±1.18)	Stage IV(3.75±0.89)	0.61	0.38
	Stage I(4.20±0.82)	Stage II(4.28±0.93)	0.8	
	Stage I(4.20±0.82)	Stage IV(3.75±0.89)	0.2	
	Stage II(4.28±0.93)	Stage IV(3.75±0.89)	0.1	
PAAD(Stage I=21,II=147,III=3,IV=4)	Stage II(3.84±1.13)	Stage I(2.98±0.90)	0.00045	
	Stage II(3.84±1.13)	Stage IV(3.27±0.74)	0.22	
	Stage II(3.84±1.13)	Stage III(2.73±0.53)	0.06	**0.0031**
	Stage I(2.98±0.90)	Stage IV(3.27±0.74)	0.53	
	Stage I(2.98±0.90)	Stage III(2.73±0.53)	0.53	
	Stage IV(3.27±0.74)	Stage III(2.73±0.53)	0.32	
OV(Stage II=24,III=328,IV=63)	Stage IV(4.04±1.66)	Stage III(4.19±1.65)	0.5	
	Stage IV(4.04±1.66)	Stage II(4.81±1.71)	0.06	0.15
	Stage III(4.19±1.65)	Stage II(4.81±1.71)	0.1	
TGCT(Stage I=104,II=13,III=14)	Stage III(2.69±1.07)	Stage I(3.07±1.25)	0.24	
	Stage III(2.69±1.07)	Stage II(2.57±1.22)	0.79	0.26
	Stage I(3.07±1.25)	Stage II(2.57±1.22)	0.19	
SKCM(Stage II=66,III=26,IV=3)	Stage II(3.17±1.59)	Stage III(3.31±1.78)	0.73	
	Stage II(3.17±1.59)	Stage IV(3.49±0.37)	0.32	0.9
	Stage III(3.31±1.78)	Stage IV(3.49±0.37)	0.68	
UVM(Stage II=39,III=36,IV=4)	Stage III(1.97±1.32)	Stage II(1.78±0.96)	0.5	
	Stage III(1.97±1.32)	Stage IV(1.79±1.36)	0.82	0.78
	Stage II(1.78±0.96)	Stage IV(1.79±1.36)	0.99	
UCS(Stage I=22,II=5,III=20,IV=10)	Stage II(1.84±1.49)	Stage III(3.25±1.58)	0.11	
	Stage II(1.84±1.49)	Stage I(2.98±1.10)	0.17	
	Stage II(1.84±1.49)	Stage IV(1.89±1.37)	0.95	**0.03**
	Stage III(3.25±1.58)	Stage I(2.98±1.10)	0.53	
	Stage III(3.25±1.58)	Stage IV(1.89±1.37)	0.02	
	Stage I(2.98±1.10)	Stage IV(1.89±1.37)	0.04	
BLCA(Stage II=130,III=140,IV=133)	Stage III(4.17±1.45)	Stage IV(4.18±1.19)	0.96	
	Stage III(4.17±1.45)	Stage II(4.49±1.34)	0.06	0.09
	Stage IV(4.18±1.19)	Stage II(4.49±1.34)	0.05	
ACC(Stage I=9,II=36,III=15,IV=15)	Stage II(1.91±1.24)	Stage IV(3.20±1.53)	0.0087	
	Stage II(1.91±1.24)	Stage III(1.85±1.41)	0.88	
	Stage II(1.91±1.24)	Stage I(1.49±1.24)	0.38	**0.0067**
	Stage IV(3.20±1.53)	Stage III(1.85±1.41)	0.02	
	Stage IV(3.20±1.53)	Stage I(1.49±1.24)	0.0074	
	Stage III(1.85±1.41)	Stage I(1.49±1.24)	0.53	
KICH(Stage I=21,II=25,III=14,IV=6)	Stage III(1.20±1.09)	Stage II(1.25±1.18)	0.9	
	Stage III(1.20±1.09)	Stage IV(1.55±0.95)	0.49	
	Stage III(1.20±1.09)	Stage I(1.45±0.94)	0.5	0.84
	Stage II(1.25±1.18)	Stage IV(1.55±0.95)	0.52	
	Stage II(1.25±1.18)	Stage I(1.45±0.94)	0.53	
	Stage IV(1.55±0.95)	Stage I(1.45±0.94)	0.82	
CHOL(Stage I=19,II=9,IV=7)	Stage IV(2.53±1.62)	Stage I(2.81±1.21)	0.68	
	Stage IV(2.53±1.62)	Stage II(3.45±1.35)	0.25	0.35
	Stage I(2.81±1.21)	Stage II(3.45±1.35)	0.25	
DLBC(Stage I=8,II=16,III=5,IV=12)	Stage II(2.43±2.11)	Stage IV(2.27±1.45)	0.81	
	Stage II(2.43±2.11)	Stage III(2.22±0.89)	0.75	
	Stage II(2.43±2.11)	Stage I(0.90±1.37)	0.05	0.21
	Stage IV(2.27±1.45)	Stage III(2.22±0.89)	0.93	
	Stage IV(2.27±1.45)	Stage I(0.90±1.37)	0.05	
	Stage III(2.22±0.89)	Stage I(0.90±1.37)	0.06	
