# Supplementary figures and images for: Pan-cancer multi-omics profiling of OAS3 reveals its immunological and prognostic associations across human cancers
Source: PeerJ. 2026 Feb 12;14:e20805. doi: 10.7717/peerj.20805 (PMC12906706; doi:10.7717/peerj.20805)

Figure 10 I


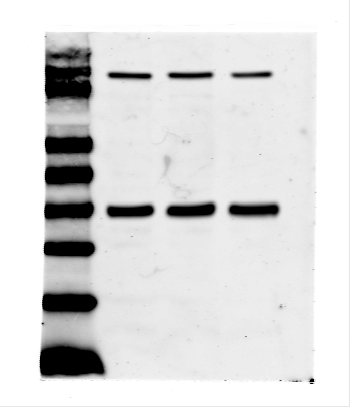


180

130

OAS3

GAPDH

33

25

KDa

95

70

55

10

17

Supplement: Supplemental Information 3 — Protein level of OAS3 in THP-1 after OAS3 knockdown [file peerj-14-20805-s003.docx]

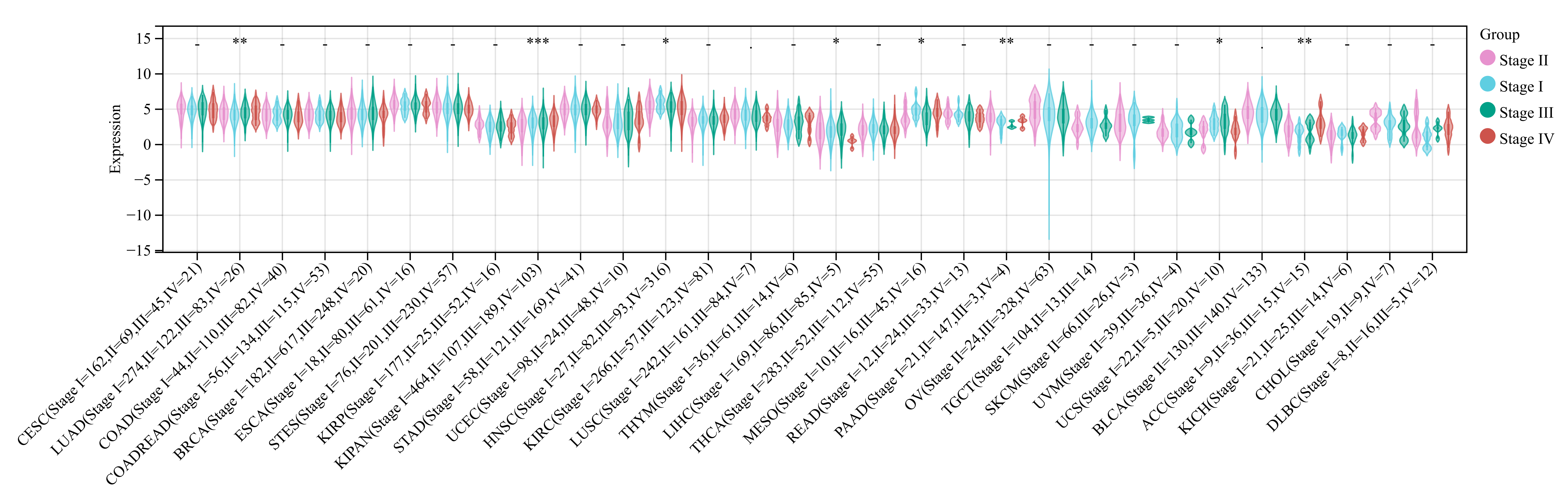

Supplement: Supplemental Information 4 [file peerj-14-20805-s004.png]

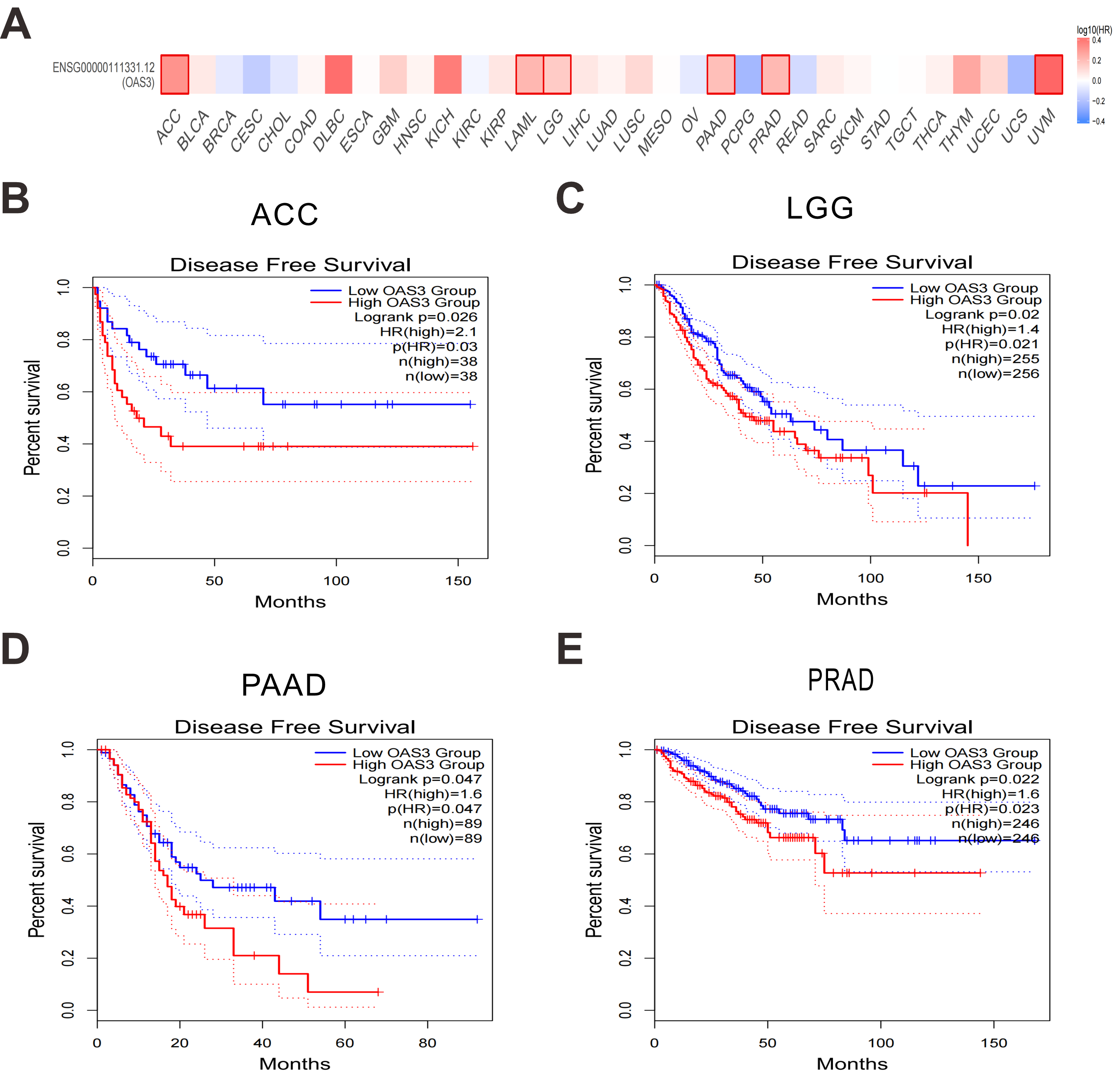

Supplement: Supplemental Information 5 — Survival map of OAS3 expression on the DFS (I-M) across various cancer types. The heatmap depicts the impact of OAS3 expression on the DFS across various cancer types (HR values). Red indicates that high OAS3 expression is associated with poorer DFS, while blue indicates better DFS. [file peerj-14-20805-s005.png]

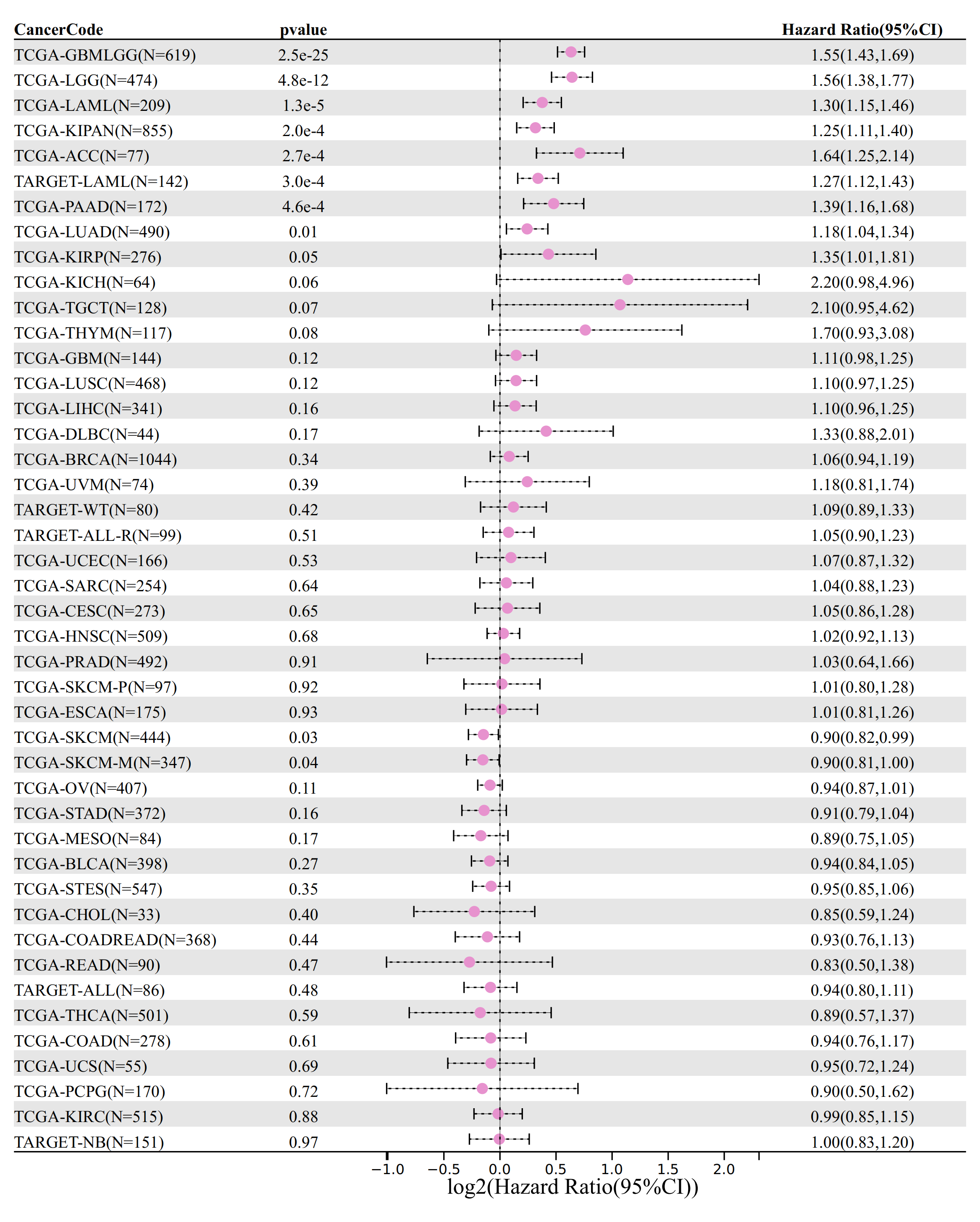

Supplement: Supplemental Information 6 — Cox regression analysis of OAS3 expression across cancer types. [file peerj-14-20805-s006.png]

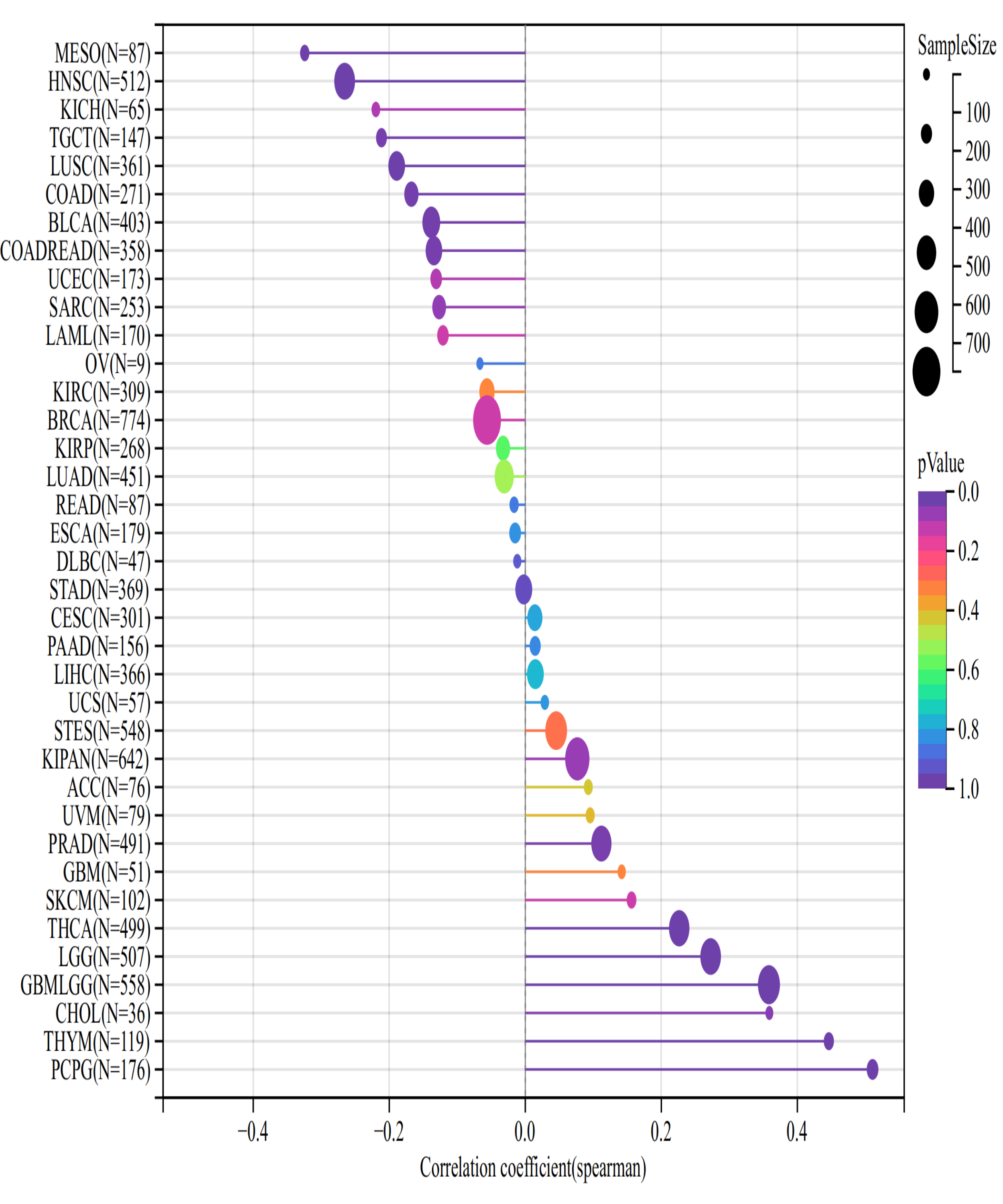

Supplement: Supplemental Information 7 [file peerj-14-20805-s007.png]

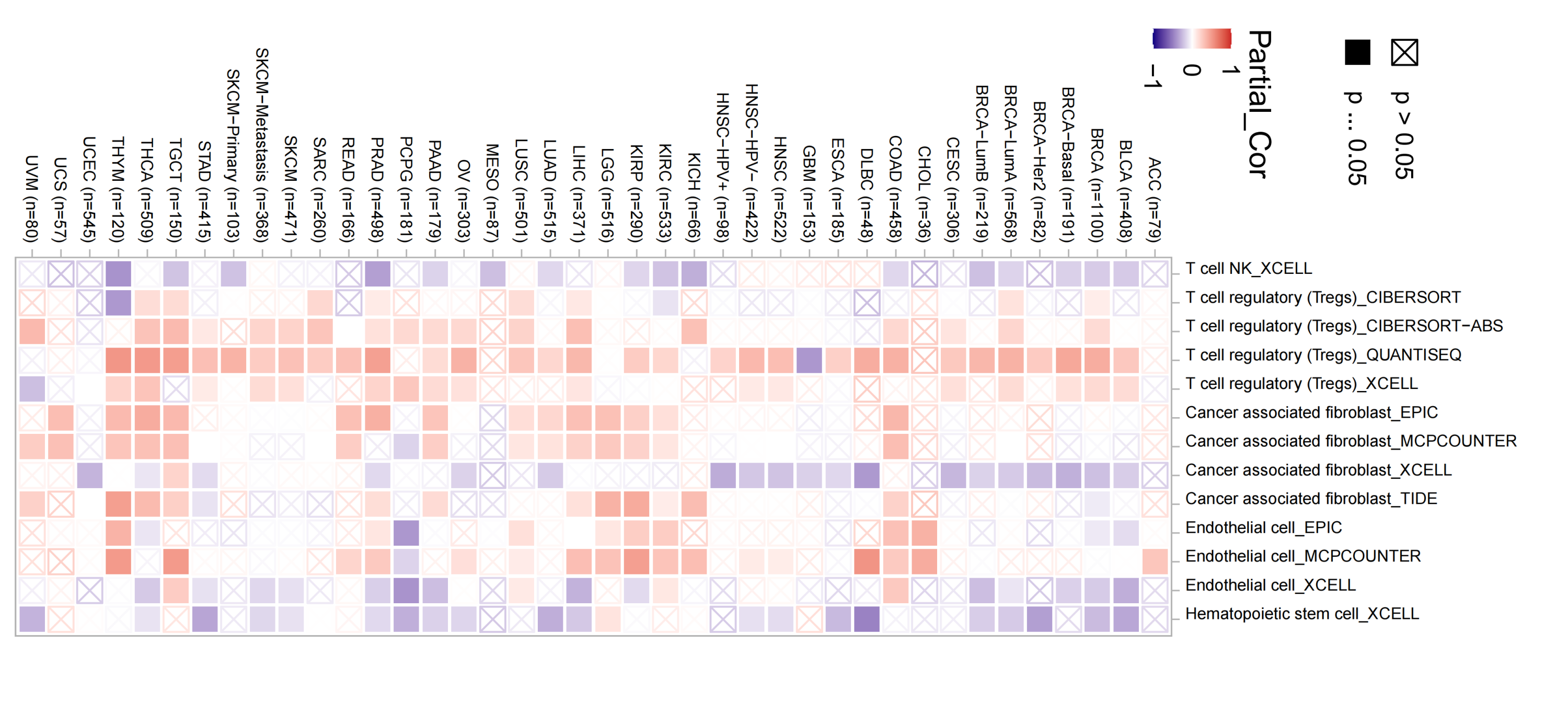

Supplement: Supplemental Information 8 — Squares marked with an ” × ” represent p-values than 0.05. [file peerj-14-20805-s008.png]

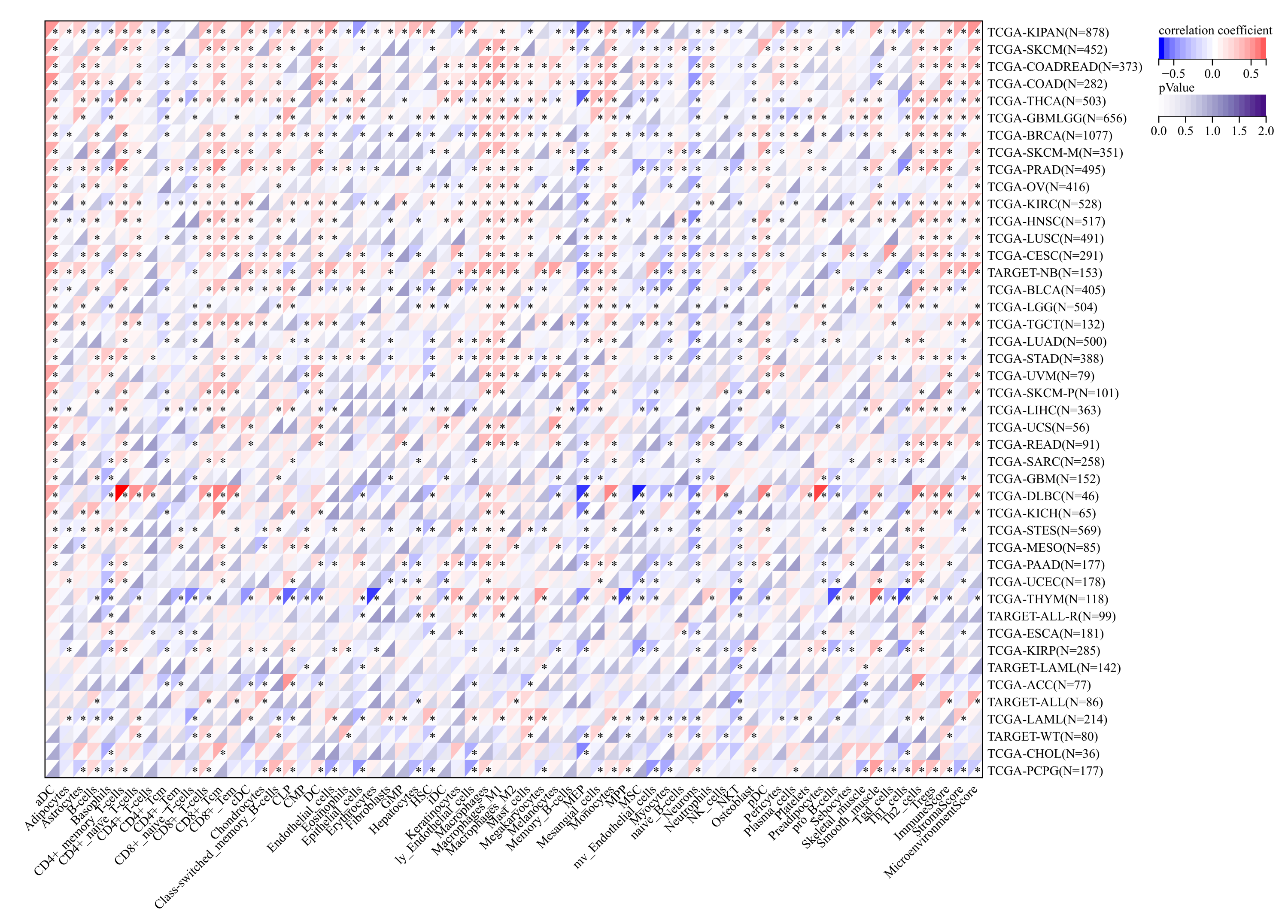

Supplement: Supplemental Information 9 [file peerj-14-20805-s009.png]

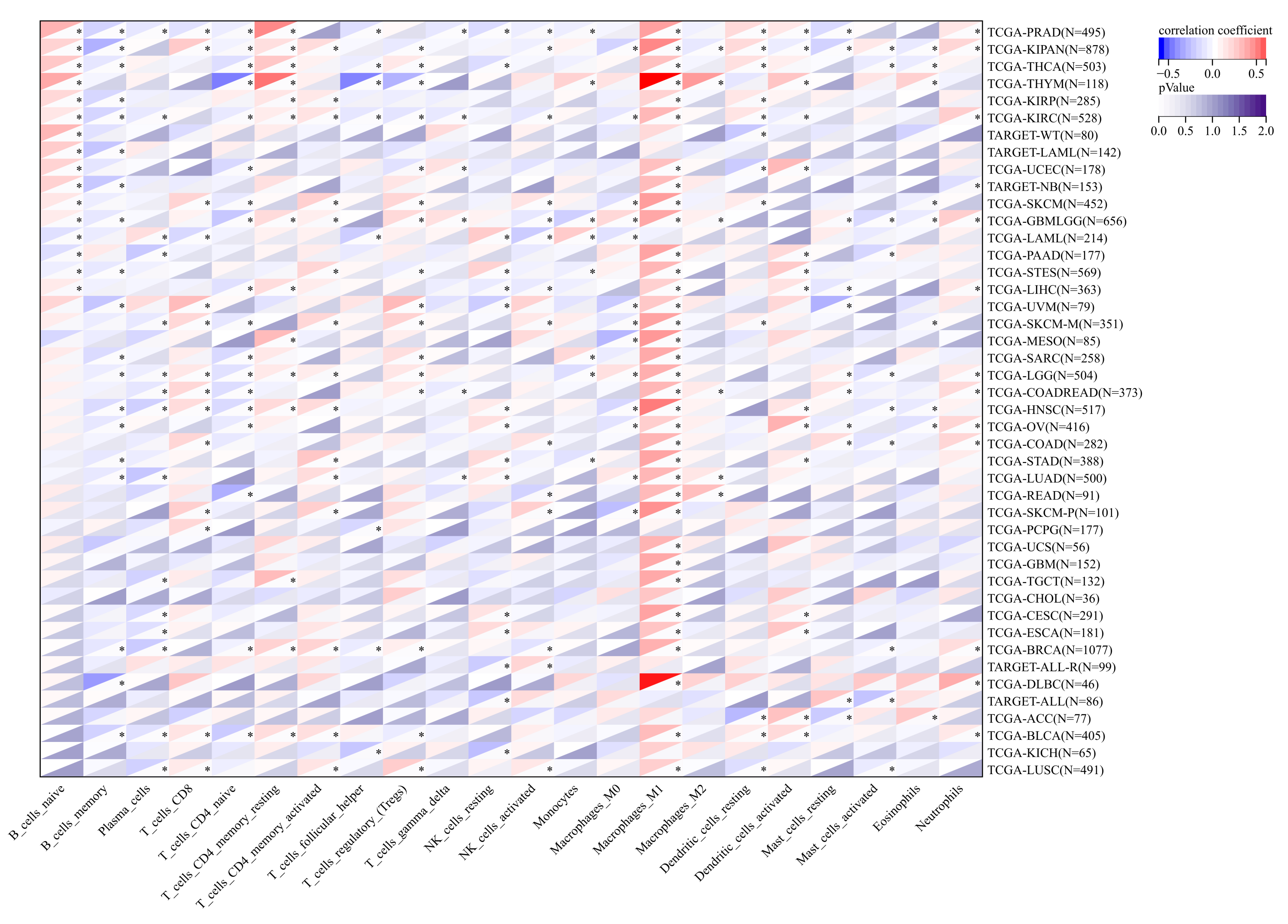

Supplement: Supplemental Information 10 [file peerj-14-20805-s010.png]

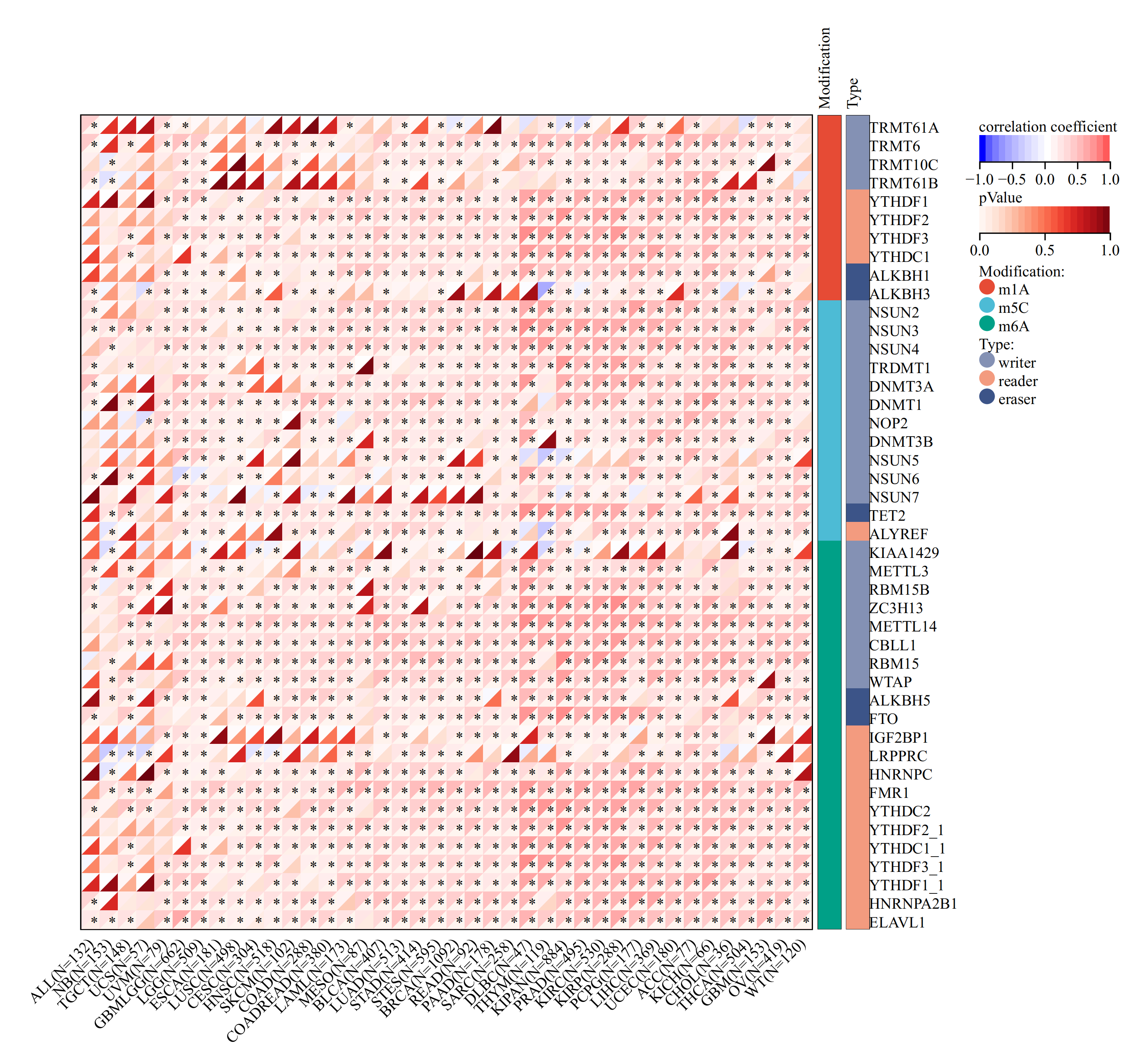

Supplement: Supplemental Information 11 [file peerj-14-20805-s011.png]

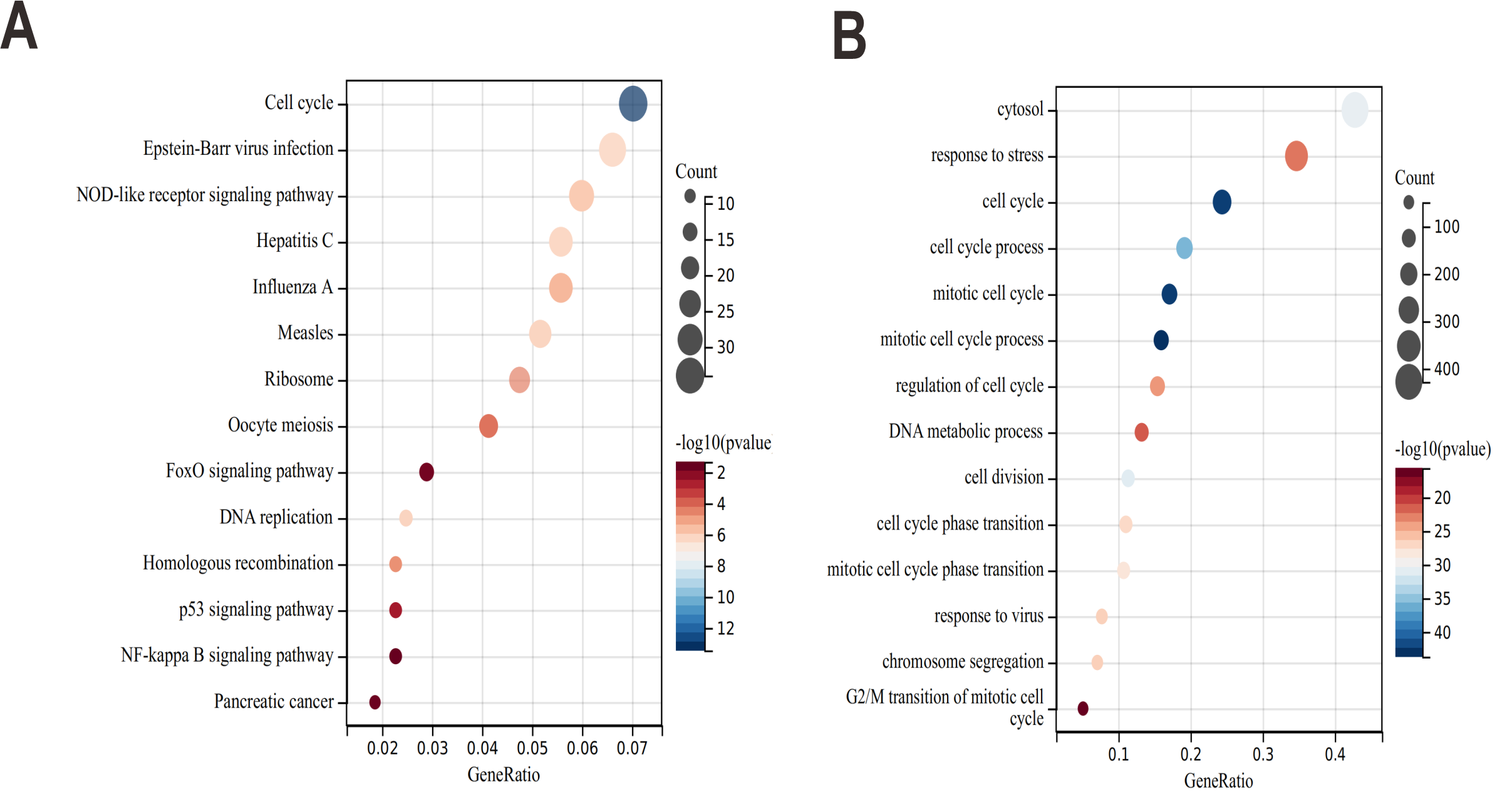

Supplement: Supplemental Information 12 — KEGG (A) and GO(B) pathway enrichment analysis of genes correlated with OAS3 in lung cancer. [file peerj-14-20805-s012.png]
